# Supplementary material for: The global impact of non-alcoholic fatty liver disease (including cirrhosis) in the elderly from 1990 to 2021 and future projections of disease burden
Source: PLoS One. 2025 Jun 25;20(6):e0325961. doi: 10.1371/journal.pone.0325961 (PMC12193573; doi:10.1371/journal.pone.0325961)
Supplement: S1 Table — (PDF) [file pone.0325961.s001.pdf]

S1 Table. Incidence of NAFLD among eldly in all countries and regions in 1990 and 2021

	1990		2021	
location	Counts	ASIR per 100,000 (95% UI)	Counts	ASIR per 100,000 (95% UI)
Afghanistan	7990（11314±5193)	900.83（1281.35±582.85)	8181(11505±5337）	1012.65（1427.10±661.26)
Albania	1321（1876±850)	530.78（757.06±340.54)	3329(4769±2128）	550.43（789.10±351.38)
Algeria	13447（19132±8647)	867.01（1240.88±556.56)	40766(58115±25816）	919.97（1315.43±581.11)
American Samoa	17（25±11)	646.40（943.07±400.28)	38(55±24）	633.10（926.72±394.30)
Andorra	29（42±19)	391.22（567.87±248.06)	83(119±53）	431.44（619.76±275.02)
Angola	2172（3058±1376)	504.48（716.53±318.65)	6924(9906±4354）	528.85（761.27±332.03)
Antigua and Barbuda	43（62±27)	638.52（907.36±406.52)	90(128±58）	660.14（935.02±422.69)
Argentina	18631（26708±11772)	443.24（636.66±279.89)	35123(50101±22271）	491.78（700.77±312.05)
Armenia	2156（3050±1377)	625.31（888.57±397.90)	3974(5697±2536）	646.32（927.71±411.06)
Australia	9314（13344±5943)	360.12（516.39±229.87)	23031(33429±14620）	400.21（579.66±254.57)
Austria	6292（9074±3995)	408.27（587.17±260.13)	10028(14366±6416）	453.27（645.93±291.18)
Azerbaijan	3666（5220±2338)	624.36（891.37±396.38)	8556(12191±5459）	640.30（918.50±406.25)
Bahamas	117（168±74)	661.90（947.68±419.85)	341(482±217）	670.43（950.57±426.62)
Bahrain	160（231±101)	835.16（1211.78±523.05)	961(1379±592）	849.04（1226.53±521.19)
Bangladesh	36908（52253±23670)	681.42（967.70±435.97)	127567(180023±81619）	740.47（1047.75±473.46)
Barbados	236（342±148)	636.03（917.00±399.61)	453(649±286）	646.33（926.74±407.95)
Belarus	8082（11585±5101)	471.93（677.75±297.66)	10767(15575±6860）	486.21（703.46±309.23)
Belgium	7825（11238±4990)	386.76（553.77±246.99)	12483(17957±8048）	431.11（617.64±278.65)
Belize	80（114±51)	700.47（996.40±449.09)	256(365±164）	722.54（1031.77±460.00)
Benin	1474（2100±939)	617.65（882.11±392.83)	3815(5458±2425）	651.97（936.05±413.63)
Bermuda	50（72±31)	627.96（907.63±398.06)	113(163±71）	634.01（910.43±399.16)
Bhutan	181（257±116)	658.17（940.45±420.63)	528(753±336）	719.99（1028.75±457.56)
Bolivia (Plurinational State of)	2125（3064±1345)	557.41（806.17±352.31)	6596(9579±4216）	577.53（841.09±368.41)
Bosnia and Herzegovina	2876（4123±1832)	570.17（818.09±361.96)	4921(7063±3146）	578.24（830.02±369.23)
Botswana	450（633±293)	655.46（928.89±425.32)	1214(1716±779）	697.79（991.64±445.82)
Brazil	74363（105231±47651)	687.88（976.76±440.02)	228631(324565±145231）	715.35（1016.77±454.03)
Brunei Darussalam	60（86±39)	556.13（794.88±356.33)	255(365±164）	614.33（883.94±391.72)
Bulgaria	8219（11926±5115)	474.78（690.70±295.76)	9110(13246±5745）	479.01（695.63±302.36)
Burkina Faso	2248（3201±1426)	423.60（606.54±268.02)	4806(6858±3094）	445.65（638.86±286.21)
Burundi	1619（2300±1039)	592.64（845.51±379.73)	3472(4887±2238）	618.81（876.36±396.75)
Cabo Verde	168（241±106)	581.27（830.96±367.18)	338(489±211）	616.43（892.11±384.94)
Cambodia	3209（4571±2054)	607.09（869.32±387.33)	9715(13761±6152）	633.76（902.27±400.17)
Cameroon	3140（4502±1978)	594.88（857.64±373.94)	8910(12723±5647）	619.23（888.38±391.29)
Canada	17457（25103±11181)	411.13（591.56±263.33)	44134(63011±28680）	460.69（657.21±299.76)
Central African Republic	692（979±443)	529.29（756.14±337.52)	1300(1836±841）	548.07（780.77±352.05)
Chad	1997（2833±1280)	588.43（837.32±376.77)	4064(5786±2603）	623.80（891.60±398.74)
Chile	5558（7990±3507)	449.48（647.34±283.45)	16630(24033±10405）	501.06（724.37±313.47)
China	636123（899548±406516)	613.44（872.27±391.21)	1831634(2602902±1167153）	670.18（954.78±426.93)
Colombia	13506（19188±8659)	651.67（928.14±416.73)	47565(67731±30356）	679.46（967.79±433.74)
Comoros	170（240±107)	716.60（1018.69±453.48)	432(615±274）	750.75（1072.57±475.74)
Congo	641（907±405)	495.12（707.37±311.48)	1532(2203±973）	515.13（744.15±325.97)
Cook Islands	9（13±6)	582.69（843.66±367.49)	20(29±12）	578.37（834.78±357.98)
Costa Rica	1334（1902±851)	634.43（905.37±404.45)	4625(6623±2943）	655.19（938.83±416.86)
Coted'Ivoire	2335（3345±1470)	531.93（766.40±334.12)	6867(9904±4351）	553.76（802.54±350.13)
Croatia	3549（5152±2234)	437.17（635.98±274.55)	5150(7469±3247）	439.35（635.50±277.32)
Cuba	8142（11594±5246)	636.26（906.20±409.72)	15755(22731±10013）	658.95（949.24±419.52)
Cyprus	489（699±311)	448.82（643.09±284.63)	1345(1918±864）	496.23（707.63±318.63)
Czechia	7313（10705±4612)	396.24（579.56±250.06)	11034(16014±6906）	400.27（580.26±250.93)
Democratic People's Republic of Korea	11458（16302±7392)	608.55（869.96±391.31)	26021(36963±16699）	646.97（920.59±414.64)
Democratic Republic of the Congo	10310（14694±6582)	551.67（792.32±350.73)	23582(33082±15112）	572.75（809.35±364.27)
Denmark	3240（4655±2087)	312.25（447.76±201.39)	5129(7327±3287）	345.55（491.99±222.52)
Djibouti	88（124±56)	614.81（873.52±392.82)	463(657±300）	656.79（939.20±422.44)
Dominica	49（71±31)	638.35（915.09±406.02)	71(103±46）	647.59（933.50±412.70)
Dominican Republic	2853（4063±1840)	647.37（923.46±416.92)	8258(11721±5298）	678.93（964.16±435.48)
Ecuador	4377（6304±2765)	700.21（1009.61±441.90)	14507(20981±9161）	713.12（1031.71±450.20)
Egypt	27301（40323±16979)	838.89（1241.46±519.93)	67599(97782±41881）	822.26（1195.58±509.00)
El Salvador	2398（3406±1533)	671.32（954.61±428.73)	5313(7570±3385）	703.07（999.87±448.62)
Equatorial Guinea	115（165±74)	511.79（741.85±327.68)	307(449±193）	557.46（816.22±348.78)
Eritrea	654（920±424)	587.11（833.45±377.92)	1823(2563±1182）	618.82（875.61±399.47)
Estonia	1115（1619±708)	416.01（603.36±263.92)	1457(2085±917）	422.51（602.34±266.33)
Eswatini	208（296±132)	640.69（918.96±406.91)	444(629±283）	668.12（952.39±424.99)
Ethiopia	12812（18187±8155)	558.29（798.60±354.57)	28748(40870±18420）	587.30（837.31±375.53)
Fiji	275（388±177)	700.84（995.68±449.22)	706(1001±449）	713.28（1017.56±452.30)
Finland	3455（4928±2233)	369.73（526.54±239.07)	6766(9706±4365）	416.37（594.65±269.58)
France	40716（59186±25951)	381.18（551.95±243.04)	72552(104646±46056）	420.41（603.21±267.65)
Gabon	367（528±229)	510.83（737.57±318.65)	683(1006±428）	537.96（795.20±335.92)
Gambia	242（346±155)	603.71（869.59±386.45)	702(1001±448）	631.15（902.96±401.83)
Georgia	5185（7512±3299)	625.60（907.41±396.77)	5195(7464±3301）	638.51（916.88±405.62)
Germany	60738（87484±38529)	377.08（540.61±239.89)	98920(142348±63349）	418.66（598.14±268.82)
Ghana	3935（5601±2485)	545.31（780.27±343.79)	11711(16801±7410）	595.47（858.94±375.71)
Greece	8955（12904±5636)	448.32（645.73±282.06)	13939(20100±8809）	491.44（704.00±311.35)
Greenland	17（25±11)	478.32（685.32±305.12)	49(69±31）	535.65（757.80±342.90)
Grenada	59（84±37)	633.76（902.69±405.33)	93(133±60）	656.90（937.87±420.54)
Guam	56（79±36)	598.80（853.41±380.35)	170(244±108）	607.85（872.95±387.13)
Guatemala	2989（4201±1930)	722.36（1020.32±464.92)	10337(14592±6698）	763.22（1079.26±493.95)
Guinea	2785（3937±1778)	663.78（941.69±423.32)	4582(6461±2934）	690.17（977.40±440.84)
Guinea-Bissau	292（412±188)	624.71（886.45±400.83)	526(747±339）	656.36（937.08±420.59)
Guyana	280（398±178)	622.09（884.99±394.20)	530(760±337）	650.80（937.91±412.81)
Haiti	2563（3612±1667)	657.14（933.13±425.91)	5721(7962±3744）	681.93（955.53±444.26)
Honduras	1742（2457±1116)	723.18（1022.86±462.93)	5876(8255±3817）	758.58（1069.10±491.63)
Hungary	9463（13773±5889)	477.54（695.12±297.85)	12337(17909±7766）	479.63（695.71±302.87)
Iceland	149（215±93)	404.08（584.38±254.07)	334(475±213）	443.76（629.88±283.43)
India	361078（505287±232067)	675.80（952.19±432.77)	1096309(1531859±703201）	736.88（1034.24±471.81)
Indonesia	81143（113835±52309)	711.11（1003.57±457.07)	221062(311337±141652）	740.35（1049.23±472.79)
Iran (Islamic Republic of)	30492（42912±19603)	913.04（1296.27±583.80)	89031(126383±56408）	926.19（1317.72±585.69)
Iraq	7998（11443±5041)	873.19（1250.82±549.86)	26260(37319±16679）	935.90（1335.06±592.93)
Ireland	2414（3507±1527)	447.80（650.59±283.18)	4938(7136±3127）	491.59（709.55±311.61)
Israel	3478（4974±2201)	545.54（779.92±345.90)	9173(13185±5799）	584.52（839.72±369.81)
Italy	59832（85620±38140)	507.84（725.64±324.06)	90999(130375±57850）	532.89（759.40±340.67)
Jamaica	1474（2107±949)	648.01（925.27±417.94)	2608(3746±1644）	667.51（958.51±420.88)
Japan	88031（125410±56570)	403.51（575.37±259.21)	189048(272266±121334）	435.58（620.70±281.09)
Jordan	1272（1820±808)	860.86（1235.12±545.14)	7643(10926±4772）	883.48（1268.20±550.71)
Kazakhstan	9056（12935±5764)	573.38（822.41±363.64)	13791(19764±8710）	594.91（856.31±374.08)
Kenya	5938（8403±3815)	619.22（879.62±396.94)	17614(25024±11289）	654.95（935.61±418.32)
Kiribati	31（44±20)	710.16（1013.76±453.09)	66(93±43）	737.23（1046.90±472.93)
Kuwait	519（754±322)	842.04（1228.14±522.72)	2596(3733±1603）	848.85（1225.32±522.80)
Kyrgyzstan	2508（3578±1592)	664.07（948.40±420.51)	4019(5690±2591）	678.87（965.04±434.93)
Lao People's Democratic Republic	1368（1937±874)	548.86（781.43±349.90)	3157(4518±2011）	580.76（834.70±368.67)
Latvia	1971（2865±1252)	421.31（612.04±267.65)	2216(3226±1394）	432.09（626.37±272.43)
Lebanon	2339（3359±1494)	849.26（1223.15±541.18)	6390(9135±4021）	903.75（1288.38±569.87)
Lesotho	628（889±408)	612.23（869.96±397.30)	865(1227±565）	646.26（923.14±420.37)
Liberia	896（1270±566)	621.03（883.96±392.01)	1469(2084±948）	652.53（929.50±419.52)
Libya	1989（2864±1267)	892.75（1286.98±567.76)	5404(7759±3424）	940.61（1352.61±594.73)
Lithuania	2330（3373±1468)	399.02（577.12±251.03)	3053(4426±1925）	410.98（593.66±259.46)
Luxembourg	261（379±164)	367.84（533.91±232.25)	534(771±341）	407.28（587.00±260.57)
Madagascar	3594（5079±2303)	594.61（844.53±379.77)	7669(10768±4921）	617.79（874.10±394.98)
Malawi	2960（4243±1892)	635.38（916.30±404.78)	5686(8164±3603）	662.01（955.09±418.72)
Malaysia	7529（10882±4788)	699.90（1012.42±444.31)	26502(38488±16746）	724.36（1053.99±456.91)
Maldives	63（89±41)	607.86（866.41±388.87)	236(336±150）	643.44（916.84±406.44)
Mali	3339（4723±2151)	694.36（988.92±445.28)	7573(10584±4861）	730.48（1026.51±467.37)
Malta	260（376±165)	472.01（682.14±299.15)	672(966±425）	523.20（751.57±331.39)
Marshall Islands	12（17±8)	636.75（904.72±407.92)	28(39±18）	655.44（938.75±417.22)
Mauritania	878（1245±554)	739.64（1052.19±466.75)	2039(2875±1293）	772.44（1093.95±488.75)
Mauritius	506（722±319)	547.42（785.28±344.56)	1428(2066±896）	565.34（820.08±354.18)
Mexico	33676（47753±21238)	670.69（953.19±422.24)	107343(152168±68304）	682.53（969.20±433.70)
Micronesia (Federated States of)	39（55±25)	629.30（896.11±399.40)	62(89±40）	647.61（926.88±410.70)
Monaco	39（56±24)	426.07（618.86±266.50)	56(81±35）	462.87（668.25±293.85)
Mongolia	754（1066±481)	612.11（867.16±389.78)	1609(2280±1022）	620.80（881.71±393.21)
Montenegro	363（533±229)	464.14（680.20±292.38)	637(929±406）	470.15（685.99±299.20)
Morocco	15329（21761±9748)	880.71（1252.77±559.31)	41969(59841±26594）	950.49（1357.10±600.98)
Mozambique	3938（5539±2520)	567.15（803.18±361.21)	7572(10616±4888）	600.99（849.32±386.19)
Myanmar	19391（27233±12519)	678.76（958.84±436.23)	43609(61326±28013）	711.40（1005.21±455.49)
Namibia	436（623±283)	531.26（764.17±342.93)	892(1284±578）	550.79（795.21±355.36)
Nauru	3（5±2)	614.10（874.01±390.07)	4(6±3）	653.72（922.56±415.83)
Nepal	6407（9094±4102)	590.24（842.85±376.79)	19356(27458±12320）	660.25（940.54±419.45)
Netherlands	9372（13525±5949)	364.14（524.98±231.34)	17840(25799±11432）	394.49（569.88±253.15)
New Zealand	1979（2840±1265)	383.00（549.73±245.11)	4648(6639±2959）	424.81（605.59±270.68)
Nicaragua	1166（1647±749)	655.86（928.74±420.66)	4025(5731±2594）	684.76（976.57±440.63)
Niger	2109（2999±1359)	650.87（932.22±417.96)	6605(9406±4255）	679.45（971.94±436.74)
Nigeria	33834（47919±21618)	637.42（906.24±406.60)	69091(97703±44096）	672.08（954.68±427.76)
Niue	2（2±1)	616.00（887.33±390.05)	2(3±1）	620.94（897.40±394.36)
North Macedonia	1165（1685±748)	490.90（711.80±314.14)	2267(3245±1447）	497.28（714.39±316.67)
Northern Mariana Islands	10（14±6)	612.76（885.40±381.14)	41(60±26）	624.59（908.51±391.40)
Norway	3442（4948±2193)	385.49（552.96±245.65)	5355(7711±3423）	423.81（609.22±271.45)
Oman	630（895±405)	860.27（1226.05±551.68)	1897(2711±1193）	895.94（1284.69±562.15)
Pakistan	49455（69698±31658)	736.79（1041.53±471.11)	109865(153215±70796）	785.79（1101.59±504.84)
Palau	8（11±5)	633.66（902.34±402.18)	19(28±12）	638.66（924.98±399.08)
Palestine	931（1325±595)	869.55（1241.10±554.87)	2788(3963±1798）	936.05（1336.24±601.30)
Panama	1142（1621±731)	639.39（908.84±409.18)	3673(5215±2333）	672.90（954.88±427.65)
Papua New Guinea	1311（1843±845)	615.20（873.63±394.45)	3490(4873±2277）	628.99（885.50±408.14)
Paraguay	1524（2175±959)	570.18（815.18±358.86)	4295(6088±2739）	592.80（841.97±377.82)
Peru	7944（11331±5043)	567.49（810.93±359.96)	23910(34165±15311）	586.82（838.57±375.85)
Philippines	20187（28864±12776)	596.74（856.28±376.85)	62136(88932±39387）	620.15（891.77±392.13)
Poland	27771（39795±17608)	480.72（689.40±304.82)	48132(68967±30542）	491.09（704.19±311.64)
Portugal	9043（13000±5768)	481.67（693.32±307.17)	16002(23117±10208）	531.35（764.80±339.59)
Puerto Rico	3153（4558±1997)	679.66（982.40±430.89)	6186(8917±3853）	686.63（986.48±429.67)
Qatar	76（111±47)	815.38（1196.43±505.21)	723(1053±443）	796.55（1162.38±485.20)
Republic of Korea	15464（22316±9863)	454.79（659.34±288.88)	63274(91214±40130）	502.47（724.95±318.45)
Republic of Moldova	2588（3768±1629)	448.76（654.14±281.61)	3774(5514±2352）	461.88（675.48±287.51)
Romania	17027（24554±10868)	460.11（664.64±292.84)	23501(34015±14831）	475.64（687.15±300.46)
Russian Federation	128021（182729±81985)	537.32（767.26±343.53)	182749(260626±116645）	547.13（781.94±348.55)
Rwanda	1756（2514±1118)	520.17（747.86±329.97)	4157(5886±2670）	548.99（782.42±351.34)
Saint Kitts and Nevis	35（50±22)	661.27（951.21±424.24)	65(94±42）	684.27（986.96±436.03)
Saint Lucia	71（100±45)	617.02（876.76±393.87)	192(274±122）	632.53（904.04±399.72)
Saint Vincent and the Grenadines	60（86±38)	645.40（928.52±408.78)	124(179±79）	656.32（949.16±419.23)
Samoa	65（94±41)	609.93（885.64±384.05)	110(159±70）	623.58（901.45±397.31)
San Marino	18（27±12)	400.91（582.07±253.13)	38(56±24）	433.79（630.73±275.44)
Sao Tome and Principe	48（69±30)	574.04（827.53±363.14)	77(111±49）	599.94（871.27±379.70)
Saudi Arabia	5626（8037±3527)	878.65（1259.68±549.63)	17450(24945±10844）	868.56（1249.34±537.00)
Senegal	2870（4075±1842)	736.60（1049.79±472.02)	7115(10075±4567）	766.81（1089.99±490.59)
Serbia	8793（12527±5585)	560.76（803.69±354.94)	12768(18364±8086）	570.98（821.09±362.28)
Seychelles	44（63±28)	607.61（873.89±380.03)	91(131±57）	613.72（887.66±380.51)
Sierra Leone	1453（2076±924)	586.56（839.76±372.92)	2607(3717±1667）	607.38（868.95±387.23)
Singapore	1394（2001±888)	538.77（776.05±342.40)	6754(9644±4259）	595.43（852.21±375.05)
Slovakia	3164（4538±1997)	398.14（571.69±251.59)	5410(7759±3425）	417.96（599.98±264.79)
Slovenia	1230（1767±781)	388.93（558.00±247.04)	2252(3260±1415）	397.08（573.00±250.09)
Solomon Islands	100（143±65)	626.06（897.01±402.48)	250(354±161）	658.22（935.46±422.33)
Somalia	1698（2392±1092)	719.82（1019.27±461.04)	5131(7236±3344）	743.96（1057.44±481.74)
South Africa	16281（22881±10457)	654.30（922.81±419.42)	38182(54101±24381）	661.45（940.40±421.28)
South Sudan	2056（2921±1315)	653.53（932.70±417.17)	2858(4040±1824）	685.63（974.64±436.36)
Spain	35802（51298±22750)	489.92（701.83±311.45)	62658(90278±39701）	536.50（768.97±340.79)
Sri Lanka	7871（11180±5035)	611.42（872.73±390.04)	23773(33856±15039）	647.20（924.60±409.17)
Sudan	8953（12330±5994)	810.94（1123.28±541.04)	21030(29009±14002）	965.01（1337.18±640.46)
Suriname	198（283±126)	643.69（920.34±409.15)	532(765±340）	668.90（962.55±426.06)
Sweden	7285（10459±4665)	375.00（535.18±240.54)	10857(15665±6898）	413.66（593.86±263.84)
Switzerland	4574（6655±2916)	351.09（509.03±224.33)	8461(12197±5385）	386.89（554.68±247.49)
Syrian Arab Republic	5513（7795±3531)	884.22（1255.49±564.11)	16123(22845±10180）	947.20（1347.49±596.32)
Taiwan (Province of China)	12296（17743±7759)	588.99（852.56±371.21)	37230(53643±23665）	645.62（931.23±410.27)
Tajikistan	2148（3042±1375)	650.44（923.27±415.72)	4666(6536±3005）	671.10（949.48±429.81)
Thailand	24530（34860±15762)	592.86（846.01±379.92)	90060(129799±56871）	624.50（901.52±393.95)
Timor-Leste	183（257±118)	638.11（902.53±407.83)	744(1050±483）	670.48（949.93±434.28)
Togo	833（1184±534)	603.20（861.01±385.15)	2716(3856±1742）	635.75（909.17±405.89)
Tokelau	1（2±1)	621.51（885.49±397.28)	1(2±1）	646.60（921.74±412.03)
Tonga	43（62±27)	628.99（900.05±396.80)	62(89±39）	640.61（919.21±405.47)
Trinidad and Tobago	637（916±405)	607.40（874.89±385.64)	1624(2355±1017）	616.76（896.01±385.92)
Tunisia	5339（7579±3435)	840.79（1198.90±539.82)	15804(22386±10070）	897.15（1274.56±570.72)
Turkey	35014（50039±22380)	834.19（1194.95±532.26)	107599(154579±68217）	883.60（1272.42±559.47)
Turkmenistan	1412（2034±899)	607.89（877.94±385.71)	3109(4485±1942）	626.82（904.97±390.64)
Tuvalu	6（8±4)	638.46（915.43±407.96)	9(13±6）	665.84（944.16±425.65)
Uganda	4143（5892±2647)	548.13（783.06±349.28)	9591(13705±6065）	584.37（837.66±368.68)
Ukraine	50806（72151±31929)	523.48（745.05±328.34)	56946(81975±35566）	535.21（770.21±333.70)
United Arab Emirates	285（411±179)	795.58（1149.89±498.91)	3027(4403±1861）	739.78（1083.58±454.20)
United Kingdom	44118（62826±28252)	373.49（530.43±239.64)	64860(92402±41375）	406.70（577.01±260.14)
United Republic of Tanzania	7736（10922±4947)	590.63（839.70±377.01)	17917(25281±11382）	618.37（876.09±392.70)
United States of America	57（82±36)	490.47（696.58±314.39)	444693(629347±285043）	567.26（802.80±363.90)
United States Virgin Islands	205496（292125±131540)	566.08（817.56±355.03)	143(206±90）	571.44（825.16±359.43)
Uruguay	2391（3429±1521)	461.58（661.81±293.66)	3560(5105±2269）	513.24（731.96±327.91)
Uzbekistan	9054（12969±5808)	650.38（932.42±415.59)	21628(31091±13840）	669.49（964.59±427.14)
Vanuatu	53（74±34)	713.74（1005.79±459.26)	153(215±100）	742.72（1046.80±480.33)
Venezuela (Bolivarian Republic of)	7445（10631±4767)	652.29（933.17±416.96)	26315(37241±16831）	674.96（958.10±431.23)
Viet Nam	29729（42202±19146)	582.51（829.26±374.49)	75770(107137±48783）	602.88（855.58±386.62)
Yemen	5148（7255±3313)	882.98（1251.85±566.57)	16320(22578±10622）	990.94（1379.18±642.70)
Zambia	1947（2788±1249)	605.04（869.31±387.11)	4786(6793±3051）	636.25（907.04±404.55)
Zimbabwe	3033（4255±1954)	619.16（873.94±397.78)	5230(7313±3424）	642.55（905.15±418.16)
