# Supplementary material for: The global impact of non-alcoholic fatty liver disease (including cirrhosis) in the elderly from 1990 to 2021 and future projections of disease burden
Source: PLoS One. 2025 Jun 25;20(6):e0325961. doi: 10.1371/journal.pone.0325961 (PMC12193573; doi:10.1371/journal.pone.0325961)
Supplement: S2 Table — (PDF) [file pone.0325961.s002.pdf]

S2 Table. Prevalence of NAFLD among eldly in all countries and regions in 1990 and 2021

	1990		2021	
location	Counts	ASPR per 100,000 (95% UI)	Counts	ASPR per 100,000 (95% UI)
Afghanistan	354975（447240±275024)	42425.82（53347.83±32956.31)	409896（506924±322617)	49911.69（61922.87±39096.47)
Albania	79128（99602±60909)	32462.20（40860.79±25003.87)	207497（258011±160022)	34443.83（42860.04±26555.40)
Algeria	689760（851210±541871)	46129.86（57013.83±36194.30)	2467243（2943419±1980126)	58059.48（69248.85±46636.24)
American Samoa	1066（1310±834)	43680.99（53594.94±34285.24)	2790（3367±2216)	49603.53（59821.81±39497.37)
Andorra	1399（1799±1064)	18955.64（24389.44±14405.75)	4705（5919±3647)	24415.42（30687.61±18950.34)
Angola	99182（126859±75174)	24689.54（31564.56±18770.91)	312777（401423±236104)	25587.53（32826.96±19366.70)
Antigua and Barbuda	2512（3148±1934)	36069.14（45255.24±27687.47)	5112（6382±3969)	38433.38（47955.81±29874.36)
Argentina	771332（998964±574604)	18577.63（24082.68±13833.29)	1735716（2208037±1332585)	23983.11（30519.51±18394.64)
Armenia	116221（147147±89455)	35328.02（44544.45±27356.72)	238017（296421±185678)	39888.40（49591.56±31179.55)
Australia	436468（559348±332297)	16863.65（21634.70±12825.72)	1302980（1630002±1008786)	22005.37（27547.04±17019.70)
Austria	282964（363382±213752)	17891.67（23004.91±13502.48)	560501（707157±434392)	24185.65（30521.57±18723.94)
Azerbaijan	194094（245404±148782)	34062.70（42944.71±26216.09)	478827（595488±372100)	38925.72（48265.16±30442.39)
Bahamas	6149（7705±4782)	34953.74（43817.58±27168.27)	19569（24322±15216)	40246.73（49890.88±31355.96)
Bahrain	8444（10423±6661)	48108.75（59238.58±38056.72)	58354（69583±47204)	60245.23（71411.80±49124.95)
Bangladesh	1579149（2022826±1207457)	29639.32（37948.02±22683.85)	5755083（7310010±4413827)	33902.16（43083.02±25998.60)
Barbados	14301（17724±11119)	36509.43（45374.88±28266.17)	27387（33812±21388)	39371.10（48600.08±30741.01)
Belarus	445675（564487±337483)	26487.19（33543.10±20066.50)	625607（787909±478210)	28795.48（36205.94±22049.10)
Belgium	362272（468321±272191)	17630.28（22805.47±13250.68)	684199（864494±528601)	22748.88（28727.89±17581.69)
Belize	4261（5316±3296)	37456.60（46723.61±28995.75)	14847（18260±11711)	43606.44（53534.75±34484.17)
Benin	69794（88822±53769)	29711.14（37821.29±22885.24)	192045（244425±147822)	34067.55（43286.91±26273.02)
Bermuda	2938（3636±2295)	37618.32（46579.39±29392.98)	7617（9362±6014)	41921.32（51552.64±33089.53)
Bhutan	7539（9584±5740)	29027.90（36883.69±22138.05)	25235（31800±19380)	34841.77（43905.18±26760.73)
Bolivia (Plurinational State of)	104915（134329±80343)	28468.22（36395.06±21826.27)	360550（452744±279002)	32694.88（41054.17±25304.99)
Bosnia and Herzegovina	157447（198578±121353)	32518.16（40950.54±25215.06)	311379（385748±242155)	36677.46（45457.08±28488.55)
Botswana	20551（26172±15754)	31568.91（40161.81±24236.99)	60400（75806±46772)	36786.25（46135.98±28509.60)
Brazil	3866533（4831147±3009406)	36928.52（46104.57±28769.62)	12880676（15817216±10145949)	40906.03（50211.47±32241.77)
Brunei Darussalam	2296（2928±1743)	21550.91（27489.29±16369.34)	10480（13226±8035)	26711.13（33719.54±20518.73)
Bulgaria	488081（617845±374870)	28703.55（36360.93±22054.05)	581326（731180±451967)	29624.62（37335.38±22962.72)
Burkina Faso	104414（133933±78838)	20485.93（26286.69±15466.45)	232970（297475±177069)	22353.69（28549.15±16990.63)
Burundi	70537（90162±53954)	26360.02（33745.23±20155.04)	151192（192007±115689)	28619.56（36291.97±21954.75)
Cabo Verde	9036（11489±6957)	30450.72（38749.25±23409.22)	18152（22928±13933)	34410.98（43339.29±26530.37)
Cambodia	158021（200438±121411)	31533.54（39974.79±24240.19)	472106（598151±361707)	32570.57（41230.26±24981.79)
Cameroon	148273（188664±113042)	29814.03（37875.08±22796.79)	449846（569395±346003)	33161.52（41897.01±25571.90)
Canada	746301（959061±560318)	17544.21（22564.07±13159.09)	2022553（2598149±1548016)	20868.45（26818.03±15966.43)
Central African Republic	27697（35646±20808)	22914.19（29498.95±17246.34)	54933（71132±41270)	25145.57（32518.53±18971.44)
Chad	92849（119552±71087)	27591.53（35566.06±21087.33)	184527（236472±139719)	29517.33（37783.53±22381.39)
Chile	248191（317435±186818)	20346.76（26036.42±15319.70)	866188（1094412±666287)	26121.09（33000.94±20099.38)
China	29907932（37798695±23010341)	29956.62（37837.58±23061.06)	98908180（123669334±77307490)	36594.82（45781.24±28537.51)
Colombia	702947（889231±541017)	34953.52（44132.91±26951.55)	2777054（3453802±2166886)	40003.65（49697.74±31257.87)
Comoros	7880（9953±6080)	35005.39（44225.67±27021.06)	20982（26344±16163)	37599.39（47198.72±28995.08)
Congo	28423（36580±21484)	23268.41（29928.29±17619.74)	73167（93229±55568)	26262.50（33445.81±19993.54)
Cook Islands	581（719±452)	39916.31（49326.71±31090.79)	1481（1807±1172)	43562.09（53143.20±34494.72)
Costa Rica	74703（93605±57979)	35842.41（44897.71±27836.96)	283589（350180±222683)	40735.48（50248.72±32015.22)
Coted'Ivoire	107205（137557±81511)	26322.11（33673.77±20094.70)	336302（429877±254027)	28882.64（36851.80±21911.23)
Croatia	202246（258014±153674)	25807.33（32863.50±19630.99)	346918（434660±269337)	28907.93（36233.28±22430.06)
Cuba	461301（577209±359117)	35655.36（44678.82±27717.48)	991541（1227229±777668)	40560.95（50231.81±31800.16)
Cyprus	22553（28983±17029)	20738.00（26730.27±15600.00)	70164（89268±54212)	25448.78（32443.37±19630.41)
Czechia	442946（564025±338223)	23930.91（30498.92±18256.61)	769376（956909±599776)	26873.79（33379.98±20892.27)
Democratic People's Republic of Korea	522278（666157±398283)	29203.57（37194.15±22335.31)	1332218（1678064±1029379)	33389.87（42078.36±25806.72)
Democratic Republic of the Congo	433091（554514±328018)	24999.28（31964.48±18978.64)	1048062（1332380±794714)	27101.65（34415.93±20646.63)
Denmark	151955（195665±116318)	14207.78（18309.74±10853.96)	287016（360462±221840)	18448.43（23226.44±14240.86)
Djibouti	3672（4699±2796)	27579.33（35226.71±21106.89)	21569（27443±16606)	32796.80（41627.36±25331.42)
Dominica	2802（3522±2182)	35863.98（45170.08±27871.91)	4291（5310±3359)	40051.80（49525.13±31397.37)
Dominican Republic	151217（189228±117203)	35023.46（43835.59±27169.61)	472285（585759±368616)	39088.98（48461.24±30521.37)
Ecuador	245228（304908±191920)	39848.10（49539.20±31198.28)	904412（1108030±716834)	44619.08（54683.75±35352.18)
Egypt	1574223（1907078±1252690)	53323.92（64341.51±42633.94)	4669962（5459252±3839559)	64721.53（75251.04±53671.90)
El Salvador	126938（158948±98425)	35990.74（45035.70±27932.86)	318951（395762±249480)	41491.84（51482.98±32449.86)
Equatorial Guinea	5463（7067±4161)	25698.57（33203.13±19588.10)	15604（19886±11916)	29954.86（38097.33±22909.43)
Eritrea	26831（34481±20237)	26050.68（33412.54±19786.12)	77785（99717±59212)	27915.22（35728.59±21267.06)
Estonia	66393（84973±50357)	24791.38（31744.67±18800.35)	96126（120032±74608)	26795.38（33446.73±20797.78)
Eswatini	10011（12724±7643)	32374.58（41133.03±24744.84)	22347（28134±17181)	35792.36（45031.25±27547.96)
Ethiopia	560951（712488±426675)	25786.45（32772.69±19639.90)	1360502（1732927±1043224)	28479.39（36241.32±21836.19)
Fiji	14615（18281±11277)	39838.12（49762.75±30770.82)	42568（52509±33359)	46725.24（57567.09±36670.26)
Finland	149310（192886±111685)	15778.11（20397.65±11795.51)	343219（439618±263114)	20288.35（25999.35±15528.81)
France	1859385（2394665±1411060)	16937.89（21842.50±12846.39)	3999046（5039494±3080691)	22213.74（27994.46±17102.93)
Gabon	16914（21659±12864)	24308.46（31136.81±18501.50)	35469（44729±27090)	29916.56（37728.31±22905.27)
Gambia	11463（14519±8812)	29596.26（37500.40±22742.87)	34729（43718±26699)	32084.99（40400.48±24691.17)
Georgia	294219（368306±227902)	36489.98（45636.44±28315.29)	310287（387233±241546)	38367.39（47838.27±29890.91)
Germany	2745148（3549422±2052539)	16575.26（21431.18±12378.37)	5510620（6891966±4270580)	22030.89（27559.85±17078.07)
Ghana	180124（231094±135932)	26258.69（33612.48±19874.42)	585629（741259±450778)	31482.64（39813.86±24271.72)
Greece	414357（530540±314503)	20594.27（26393.63±15623.38)	807003（1021133±626106)	26474.78（33512.22±20512.82)
Greenland	623（812±466)	18276.06（23790.57±13751.29)	1903（2441±1452)	22338.92（28607.27±17088.22)
Grenada	3129（3958±2411)	32805.50（41532.13±25279.99)	5246（6514±4062)	37888.72（47079.54±29342.63)
Guam	3333（4162±2595)	39107.69（48671.62±30572.09)	12046（14746±9515)	44070.51（53841.73±34881.78)
Guatemala	142655（179124±110149)	36102.42（45298.32±27876.66)	551641（686534±426519)	41316.11（51419.89±31949.15)
Guinea	130086（164879±99093)	31963.10（40530.89±24353.06)	218725（276097±168302)	34079.55（42999.58±26243.80)
Guinea-Bissau	12704（16213±9706)	28588.19（36486.82±21847.59)	23541（30125±18009)	31367.28（40055.82±24039.24)
Guyana	13990（17716±10808)	32167.41（40681.72±24862.98)	27966（35065±21543)	36514.81（45721.01±28238.12)
Haiti	112348（142417±85806)	30415.68（38531.69±23266.35)	258451（326845±197717)	32677.97（41300.71±25025.65)
Honduras	85652（107444±65976)	36415.22（45685.92±28047.90)	310666（387176±240896)	41291.97（51469.08±32019.26)
Hungary	546640（691652±420839)	27575.32（34897.05±21216.37)	824664（1032172±637802)	31253.88（39173.92±24105.11)
Iceland	7499（9600±5696)	20146.35（25796.91±15295.19)	19107（23966±14853)	25112.37（31486.56±19523.20)
India	14640143（18693954±11232220)	28664.48（36513.76±22026.27)	47804102（60077254±36845438)	32986.29（41462.99±25432.45)
Indonesia	3831765（4848996±2961965)	35628.96（44945.79±27596.56)	11310520（14113785±8802840)	40451.79（50366.21±31601.84)
Iran (Islamic Republic of)	1513314（1861784±1193424)	49481.21（60625.62±39209.43)	5865276（6913620±4777456)	64012.07（75295.27±52306.94)
Iraq	427696（525225±335266)	47547.50（58342.65±37324.46)	1491864（1799425±1192610)	57059.03（68676.04±45751.65)
Ireland	113107（145136±86106)	20732.91（26631.11±15730.12)	276795（346825±215001)	26966.65（33818.85±20926.50)
Israel	162462（205961±124652)	25241.89（32058.70±19324.48)	522016（648130±402780)	32595.29（40476.22±25090.23)
Italy	2923597（3667975±2265476)	24527.45（30828.26±18989.93)	6006612（7391315±4722150)	32659.99（40167.21±25594.22)
Jamaica	81586（102106±63595)	35172.99（44076.65±27375.71)	157756（194763±123506)	40644.68（50102.15±31882.62)
Japan	3818706（4878461±2923782)	17532.97（22418.05±13422.14)	9021576（11436636±6975642)	19648.46（24864.18±15189.37)
Jordan	67116（82623±52444)	48565.81（59619.60±38131.70)	489604（582165±398748)	60650.48（71982.95±49565.27)
Kazakhstan	471861（601040±360565)	30972.81（39352.32±23726.36)	792449（990718±620613)	36047.44（45046.14±28224.27)
Kenya	274738（349254±211974)	29535.53（37497.60±22807.65)	842906（1058935±652572)	33022.92（41391.61±25630.53)
Kiribati	1585（1989±1225)	38842.43（48656.86±30036.50)	3539（4424±2748)	43976.51（54768.02±34331.40)
Kuwait	31617（38365±25037)	54896.91（66336.56±43739.51)	183591（214837±150426)	65503.53（76234.65±54101.83)
Kyrgyzstan	131760（165838±101576)	35914.84（45126.56±27765.10)	213118（267428±165313)	38257.67（47885.08±29751.31)
Lao People's Democratic Republic	65513（84125±49905)	27860.63（35721.99±21225.84)	155851（198234±119524)	30396.33（38609.14±23358.99)
Latvia	113388（145128±86124)	24255.38（31033.49±18444.96)	143268（180028±111078)	26857.32（33723.90±20840.84)
Lebanon	123062（151371±96576)	47223.27（57947.58±37178.70)	432265（513543±350753)	58499.34（69600.43±47380.68)
Lesotho	28277（36071±21417)	28150.16（35940.00±21330.89)	40995（52118±31462)	32205.93（40893.70±24703.17)
Liberia	43723（55385±33471)	31192.95（39535.44±23851.41)	74319（93768±56800)	34804.10（43868.80±26687.88)
Libya	107796（132389±84721)	50342.93（61676.11±39708.10)	332460（397174±268464)	60835.74（72488.20±49330.23)
Lithuania	137168（175489±104547)	23593.96（30167.94±18013.25)	195900（247285±151742)	25455.74（32146.06±19736.98)
Luxembourg	12020（15530±9067)	16697.77（21596.22±12595.66)	29225（36946±22404)	21930.73（27707.58±16822.83)
Madagascar	154784（196683±118187)	26798.11（34021.78±20500.94)	339589（433069±257964)	29351.45（37286.56±22428.08)
Malawi	130262（166224±99511)	29654.08（37795.38±22674.40)	266694（339923±204995)	32563.06（41444.34±25039.71)
Malaysia	394811（500887±303331)	37947.47（48042.84±29271.70)	1515582（1885608±1185680)	43206.02（53722.81±33837.75)
Maldives	3239（4133±2493)	33849.22（42982.04±26132.27)	13334（16649±10289)	37894.49（47185.35±29378.14)
Mali	148169（187780±113049)	32484.41（41128.97±24836.44)	356886（450876±275585)	36112.03（45600.75±27890.83)
Malta	12017（15440±9138)	21906.41（28168.05±16637.97)	37006（46305±28422)	27834.95（34890.81±21328.20)
Marshall Islands	643（815±496)	35080.06（44470.24±27044.18)	1494（1871±1159)	39833.05（49724.57±31023.23)
Mauritania	42046（52938±32568)	35941.27（45333.06±27780.94)	106997（133451±82842)	42070.55（52443.79±32620.47)
Mauritius	26388（33539±20334)	30227.47（38357.65±23308.10)	85256（105963±66558)	35231.17（43730.38±27526.71)
Mexico	1901860（2362026±1500784)	38817.74（48171.09±30645.48)	6512312（7983744±5172716)	42244.46（51761.23±33577.74)
Micronesia (Federated States of)	2122（2674±1644)	36224.69（45635.04±28055.70)	3540（4385±2757)	41805.91（51618.71±32797.21)
Monaco	1921（2448±1460)	20224.30（25783.07±15351.05)	3316（4184±2554)	25944.58（32759.16±19973.14)
Mongolia	38853（49168±29880)	31939.77（40466.63±24536.41)	81802（103350±62793)	33584.11（42260.52±25891.00)
Montenegro	20755（26417±15850)	27027.41（34386.61±20687.71)	41341（51795±31941)	30883.19（38733.26±23830.58)
Morocco	758730（939713±591850)	44778.81（55438.83±34980.77)	2382878（2872574±1918874)	56772.72（68301.59±45803.74)
Mozambique	175790（224532±132966)	26562.00（33888.93±20134.10)	345250（440774±263083)	28982.51（36961.39±22146.17)
Myanmar	923019（1172674±707877)	34276.40（43518.08±26333.40)	2182792（2752012±1681556)	37430.72（47123.83±28888.86)
Namibia	19993（25499±15241)	25817.97（32964.71±19663.40)	42991（54729±32538)	27610.07（35171.03±20930.52)
Nauru	174（220±133)	35874.89（45246.19±27604.65)	250（314±194)	40126.22（50126.36±31203.69)
Nepal	274863（349816±208865)	26537.51（33736.14±20190.15)	857243（1086560±659014)	30207.93（38291.76±23231.10)
Netherlands	447141（572684±339481)	17127.29（21950.02±12990.73)	1008891（1267254±777765)	21669.80（27269.07±16686.48)
New Zealand	91665（116558±69792)	17648.20（22472.31±13412.11)	248987（310071±195175)	22382.32（27885.61±17540.16)
Nicaragua	61930（77915±48180)	35661.21（44831.29±27768.56)	232150（288722±181462)	40300.85（50087.78±31532.94)
Niger	91738（116753±69984)	30018.40（38168.32±22947.49)	300405（380631±228484)	32511.25（41150.75±24773.69)
Nigeria	1566036（1975774±1205987)	30365.76（38316.76±23390.97)	3368865（4244308±2580985)	34319.89（43135.99±26376.24)
Niue	108（134±83)	38229.29（47664.89±29609.37)	121（149±95)	43514.68（53291.11±34115.04)
North Macedonia	62474（79230±47647)	27270.48（34535.20±20822.19)	142293（176922±109920)	31705.81（39494.03±24455.20)
Northern Mariana Islands	604（748±469)	42253.05（52229.90±32968.45)	2744（3353±2155)	45804.15（55810.39±36196.59)
Norway	161820（205767±123982)	17605.01（22385.65±13460.90)	294915（371286±229910)	22678.54（28554.72±17676.15)
Oman	31207（38894±24219)	44233.68（55056.58±34395.81)	115073（137848±93006)	59077.75（70515.06±48012.73)
Pakistan	2121022（2684229±1625269)	32162.54（40731.66±24648.35)	4884053（6154757±3775038)	36564.36（46011.77±28333.76)
Palau	458（574±353)	38672.59（48396.04±29884.13)	1195（1467±939)	44271.59（54290.95±34948.18)
Palestine	47647（59019±37209)	46052.94（57006.01±36023.37)	155288（188148±123966)	55734.14（67373.95±44638.65)
Panama	62347（78191±48285)	35211.02（44168.22±27278.77)	218925（272389±170701)	40000.93（49757.34±31196.88)
Papua New Guinea	61655（78960±46862)	31892.81（40623.95±24375.45)	183544（232388±140530)	35897.90（45262.65±27583.15)
Paraguay	80862（101954±62482)	30745.56（38747.96±23768.79)	245429（307732±189924)	34747.12（43494.88±26937.86)
Peru	419101（530977±320251)	30486.76（38583.80±23335.41)	1440512（1785567±1136641)	35510.46（43990.56±28037.14)
Philippines	1018529（1283781±787042)	31276.60（39389.66±24207.56)	3215068（4024708±2497807)	33756.36（42175.91±26278.57)
Poland	1628427（2041686±1262500)	28424.67（35653.45±22047.82)	3062588（3801044±2410566)	31225.47（38751.24±24557.26)
Portugal	398349（511638±302426)	21247.55（27337.59±16105.42)	886150（1116608±687504)	27798.62（35087.46±21549.42)
Puerto Rico	191675（237152±150768)	40943.35（50742.12±32154.89)	442769（537754±353847)	46613.98（56696.66±37226.29)
Qatar	4265（5185±3339)	50904.73（61606.27±40210.12)	48676（57369±39639)	63455.34（74157.98±52441.85)
Republic of Korea	612887（790585±466566)	18608.95（23989.17±14173.32)	2809903（3580324±2152540)	22512.82（28666.40±17258.32)
Republic of Moldova	136817（174788±103519)	24538.31（31346.45±18585.35)	222135（279320±170970)	27699.10（34816.94±21339.28)
Romania	976383（1239275±749639)	26804.72（34051.45±20592.44)	1484229（1852210±1156295)	29475.04（36799.63±22928.94)
Russian Federation	6884099（8726099±5326180)	29341.07（37135.02±22740.69)	10666373（13267656±8402094)	32568.75（40469.35±25646.72)
Rwanda	77490（99214±58613)	24354.53（31142.15±18435.20)	189102（242377±142497)	26582.57（33965.18±20104.67)
Saint Kitts and Nevis	1900（2375±1472)	35316.64（44291.05±27256.91)	3486（4369±2696)	39957.79（49949.45±31065.57)
Saint Lucia	3865（4889±2970)	33916.56（42963.00±26028.20)	11549（14286±9010)	38454.09（47554.34±30039.64)
Saint Vincent and the Grenadines	3250（4058±2511)	34780.35（43501.81±26826.53)	7213（8926±5665)	39016.23（48267.75±30654.83)
Samoa	3867（4825±2996)	38653.44（48152.89±29971.60)	7013（8673±5459)	41643.22（51447.94±32490.86)
San Marino	916（1169±698)	19704.25（25172.42±14997.78)	2341（2931±1817)	25050.63（31336.16±19447.45)
Sao Tome and Principe	2406（3071±1835)	29776.07（37989.06±22726.51)	3964（4999±3067)	32519.65（40960.36±25199.58)
Saudi Arabia	289787（356237±227367)	47162.03（57898.59±37124.92)	1091577（1294417±886678)	62298.89（73477.23±51018.93)
Senegal	134885（169894±103847)	35807.67（45105.12±27584.75)	352430（443050±271507)	39422.68（49508.82±30411.03)
Serbia	479757（609485±369737)	32538.32（41205.85±25147.97)	821982（1019694±644415)	36346.59（45142.27±28441.33)
Seychelles	2568（3219±1979)	35366.79（44353.55±27245.84)	5429（6744±4246)	38585.54（47837.29±30248.85)
Sierra Leone	66833（85308±50876)	27290.69（34865.90±20756.68)	124984（159110±95650)	29947.43（38107.45±22931.99)
Singapore	60166（76568±45909)	23787.93（30272.87±18173.10)	308077（389323±237777)	27771.31（35042.45±21450.76)
Slovakia	179671（228697±136823)	22725.01（28961.68±17303.13)	343590（430725±266052)	26556.75（33328.38±20518.94)
Slovenia	71403（90611±54523)	22609.85（28681.32±17285.07)	152648（188876±119133)	26312.31（32513.83±20549.79)
Solomon Islands	4905（6256±3741)	33587.63（42681.17±25750.72)	13616（17121±10566)	38324.80（48095.13±29792.10)
Somalia	69229（88462±52653)	31410.53（40044.99±23999.29)	212993（271526±162900)	33287.12（42332.45±25539.21)
South Africa	816379（1021388±630055)	33711.62（42156.55±26056.21)	2106831（2610155±1650187)	37780.78（46749.91±29638.12)
South Sudan	94802（120156±72802)	31043.64（39407.34±23795.81)	135708（171874±103988)	34632.95（43761.03±26645.69)
Spain	1597848（2045152±1216326)	21746.38（27864.36±16541.13)	3595889（4506847±2802033)	29227.76（36656.60±22759.57)
Sri Lanka	427869（537016±327991)	34494.95（43297.96±26476.71)	1313020（1633482±1024772)	36716.16（45688.40±28645.75)
Sudan	363777（460997±279767)	33521.17（42526.92±25752.16)	902109（1137393±692740)	42858.95（54014.80±32986.73)
Suriname	10073（12635±7795)	33942.99（42503.64±26331.02)	30447（37629±23844)	39272.60（48493.71±30803.11)
Sweden	354249（451584±267953)	17573.28（22430.12±13267.92)	624397（776205±484147)	22405.33（27879.29±17319.90)
Switzerland	220205（281840±166889)	16363.34（20942.74±12382.79)	481773（609305±371995)	21067.61（26627.21±16290.33)
Syrian Arab Republic	278931（345727±216375)	47277.28（58440.70±36858.99)	922451（1112846±740521)	58225.49（70032.43±46865.22)
Taiwan (Province of China)	632576（796675±487588)	31966.81（40246.95±24676.39)	2217012（2758614±1729285)	39110.03（48612.65±30524.73)
Tajikistan	107130（135675±82280)	33323.23（42093.96±25659.84)	233710（294094±178870)	36076.29（45293.24±27738.22)
Thailand	1274685（1618997±973169)	32341.29（41020.94±24779.56)	5224144（6506461±4081842)	36969.35（45978.84±28927.85)
Timor-Leste	8677（11107±6594)	32732.61（41625.92±24960.40)	37248（47136±28499)	34495.32（43672.95±26365.21)
Togo	35681（45922±27243)	26952.92（34655.29±20575.31)	125466（158609±95630)	31074.74（39262.40±23739.99)
Tokelau	67（84±52)	35718.30（44715.48±27692.83)	81（100±63)	41662.45（51729.13±32589.27)
Tonga	2592（3239±2022)	39582.82（49366.84±30950.75)	4270（5242±3342)	44384.17（54481.42±34762.68)
Trinidad and Tobago	35375（44200±27441)	33921.48（42429.94±26267.44)	98914（123216±77352)	38578.98（48003.18±30196.75)
Tunisia	282121（347406±222100)	46285.41（57002.71±36474.82)	969391（1158294±779781)	57413.57（68494.17±46294.23)
Turkey	1858139（2286340±1453374)	46368.28（56935.07±36443.57)	6707237（8008291±5402691)	56897.80（67901.50±45853.81)
Turkmenistan	75498（94682±58439)	33876.21（42445.84±26316.48)	173623（216005±134961)	37547.75（46594.54±29224.63)
Tuvalu	287（364±221)	34973.34（44165.68±26862.86)	532（663±415)	40711.50（50717.44±31801.28)
Uganda	188458（240010±142931)	25793.80（32838.23±19592.78)	449704（572589±345011)	28639.85（36411.98±21980.90)
Ukraine	2695715（3427728±2070321)	28160.86（35802.50±21633.77)	3235134（4055724±2503427)	30481.15（38211.17±23578.93)
United Arab Emirates	15865（19520±12417)	46821.29（57471.02±36801.25)	193040（227622±156769)	59772.46（69855.39±49249.43)
United Kingdom	2086018（2636774±1602736)	17223.89（21798.30±13204.10)	3839819（4732986±3027019)	22998.04（28375.83±18104.45)
United Republic of Tanzania	350527（445124±266985)	27881.48（35416.13±21239.07)	869556（1101317±670049)	31116.63（39339.83±24020.79)
United States of America	3245（4055±2503)	19978.22（25584.48±15279.66)	19705423（24758297±15338159)	24999.58（31425.35±19450.54)
United States Virgin Islands	8501852（10877746±6506682)	33234.42（41523.07±25661.79)	9630（11721±7653)	37667.96（45937.98±29879.58)
Uruguay	105188（136116±79884)	20225.36（26194.01±15353.08)	178449（226700±135225)	24846.21（31564.97±18812.29)
Uzbekistan	485567（607842±376180)	35620.44（44499.39±27689.53)	1204446（1494841±943038)	40054.94（49570.04±31513.63)
Vanuatu	2623（3298±2037)	38208.37（47982.53±29666.21)	8444（10528±6539)	43959.63（54741.74±34118.51)
Venezuela (Bolivarian Republic of)	387983（487949±299661)	34864.22（43815.59±26958.17)	1479624（1848720±1144121)	39174.72（48877.18±30337.03)
Viet Nam	1519944（1929160±1170725)	30637.35（38857.58±23617.76)	3902179（4909497±3028395)	32611.15（40971.04±25364.17)
Yemen	219749（277890±169436)	40060.78（50563.21±30943.78)	758563（943268±591285)	48749.63（60505.43±38065.83)
Zambia	86116（109054±65623)	28035.03（35498.53±21386.67)	223324（284007±171712)	31205.77（39654.75±24044.24)
Zimbabwe	135002（172309±103066)	28832.85（36728.40±22021.39)	240158（305550±184380)	31467.83（39913.12±24195.34)
