# Supplementary material for: The global impact of non-alcoholic fatty liver disease (including cirrhosis) in the elderly from 1990 to 2021 and future projections of disease burden
Source: PLoS One. 2025 Jun 25;20(6):e0325961. doi: 10.1371/journal.pone.0325961 (PMC12193573; doi:10.1371/journal.pone.0325961)
Supplement: S3 Table — (PDF) [file pone.0325961.s003.pdf]

S3 Table. Deaths of NAFLD among eldly in all countries and regions in 1990 and 2021

	1990		2021	
location	Counts	Age standardized YLDs rate per 100,000 (95% UI	Counts	Age standardized YLDs rate per 100,000 (95% UI)
Afghanistan	84（183±32)	12.27（27.75±4.46)	76（157±32)	9.78（20.51±4.03)
Albania	8（15±4)	3.78（6.88±1.87)	16（31±8)	2.90（5.41±1.34)
Algeria	61（127±25)	6.09（12.37±2.62)	272（535±123)	8.45（16.63±3.72)
American Samoa	0（0±0)	5.28（9.89±2.56)	0（1±0)	6.41（11.50±3.16)
Andorra	1（1±0)	9.73（17.98±4.64)	1（3±1)	7.31（12.45±3.67)
Angola	28（55±13)	7.72（15.65±3.47)	77（149±36)	7.13（13.92±3.31)
Antigua and Barbuda	1（1±0)	10.52（17.74±5.81)	1（2±1)	11.75（18.77±6.65)
Argentina	279（476±152)	7.03（11.95±3.81)	457（757±252)	6.27（10.40±3.45)
Armenia	13（24±7)	4.29（7.54±2.26)	56（94±31)	9.89（16.66±5.40)
Australia	81（131±47)	3.18（5.11±1.83)	220（326±134)	3.62（5.39±2.21)
Austria	178（288±99)	11.22（18.20±6.24)	221（338±131)	9.05（13.93±5.38)
Azerbaijan	67（114±35)	13.30（22.67±6.92)	196（354±94)	19.50（35.20±9.16)
Bahamas	3（5±2)	16.16（26.27±9.29)	6（10±4)	14.07（22.54±8.07)
Bahrain	3（5±1)	23.03（42.19±11.47)	13（22±6)	24.70（42.59±12.49)
Bangladesh	254（473±121)	5.10（9.56±2.43)	652（1243±315)	4.41（8.41±2.12)
Barbados	4（7±2)	10.73（17.08±6.19)	6（9±3)	8.25（13.22±4.55)
Belarus	33（56±17)	1.95（3.34±1.04)	94（164±50)	4.27（7.42±2.28)
Belgium	238（372±138)	11.53（17.96±6.72)	308（443±194)	9.86（14.28±6.18)
Belize	1（2±1)	13.24（21.56±7.34)	7（10±4)	20.09（31.28±11.56)
Benin	19（37±9)	8.60（16.85±3.90)	45（86±22)	9.10（17.23±4.38)
Bermuda	1（2±1)	17.78（26.95±10.61)	2（3±1)	9.18（14.03±5.47)
Bhutan	1（2±1)	5.22（10.87±2.17)	5（11±3)	7.97（15.28±3.67)
Bolivia (Plurinational State c	104（202±40)	30.23（58.49±11.82)	420（772±191)	40.75（74.58±18.57)
Bosnia and Herzegovina	19（34±10)	4.06（7.30±2.04)	37（64±18)	4.35（7.65±2.14)
Botswana	4（8±2)	6.78（14.64±2.74)	10（18±5)	7.07（13.16±3.38)
Brazil	454（744±262)	4.74（7.69±2.74)	1851（2810±1122)	5.96（9.03±3.62)
Brunei Darussalam	0（0±0)	2.60（4.88±1.20)	1（1±0)	2.34（4.32±1.08)
Bulgaria	75（133±40)	4.64（8.11±2.47)	120（208±63)	6.21（10.83±3.27)
Burkina Faso	29（55±14)	6.70（12.72±3.19)	67（127±31)	7.33（13.85±3.43)
Burundi	22（43±10)	9.17（17.69±4.14)	31（59±15)	7.11（13.33±3.29)
Cabo Verde	2（3±1)	5.20（11.43±2.12)	4（8±2)	8.23（15.85±3.61)
Cambodia	45（108±17)	10.12（24.99±3.73)	106（216±47)	8.50（17.57±3.67)
Cameroon	47（88±22)	10.75（20.33±5.00)	127（241±55)	10.89（20.58±4.63)
Canada	220（363±120)	5.19（8.55±2.82)	736（1151±421)	7.45（11.69±4.26)
Central African Republic	8（15±4)	6.97（14.16±3.14)	15（29±7)	7.58（15.01±3.40)
Chad	21（42±9)	6.80（13.82±2.90)	42（83±19)	7.59（15.17±3.48)
Chile	180（295±100)	14.97（24.48±8.33)	450（713±255)	13.57（21.51±7.70)
China	2374（4112±1279)	2.84（4.93±1.52)	4533（7649±2429)	1.83（3.11±0.98)
Colombia	103（170±56)	5.34（8.84±2.91)	503（801±282)	7.34（11.67±4.11)
Comoros	2（3±1)	9.46（18.09±4.47)	5（10±2)	10.42（19.88±4.69)
Congo	9（18±4)	8.78（16.83±4.04)	23（46±11)	9.43（18.42±4.26)
Cook Islands	0（0±0)	0.33（0.64±0.14)	0（0±0)	0.38（0.75±0.16)
Costa Rica	22（36±12)	10.82（17.62±6.01)	118（183±68)	17.15（26.50±9.88)
Coted'Ivoire	29（55±14)	8.57（16.34±4.06)	81（153±38)	8.16（15.50±3.84)
Croatia	49（84±26)	6.35（10.99±3.39)	65（109±36)	5.33（8.95±2.89)
Cuba	111（180±62)	8.69（14.14±4.91)	264（407±153)	10.64（16.45±6.21)
Cyprus	8（15±4)	9.89（18.11±4.75)	12（21±6)	4.78（8.24±2.46)
Czechia	73（127±39)	3.95（6.86±2.13)	138（235±76)	4.89（8.36±2.69)
Democratic People's Republ	39（80±17)	2.49（5.24±1.07)	68（142±29)	1.81（3.80±0.78)
Democratic Republic of the	82（157±39)	5.32（10.39±2.47)	168（340±74)	4.87（9.85±2.10)
Denmark	44（73±25)	4.20（6.92±2.33)	92（143±53)	5.88（9.18±3.41)
Djibouti	1（2±0)	6.96（13.80±3.20)	4（9±2)	8.42（16.81±3.61)
Dominica	1（2±1)	15.07（25.83±7.89)	2（3±1)	14.98（24.94±8.00)
Dominican Republic	77（131±41)	20.38（34.60±10.92)	260（473±122)	21.75（39.60±10.21)
Ecuador	170（263±102)	29.01（44.76±17.32)	753（1110±462)	38.21（56.15±23.50)
Egypt	1222（2136±616)	67.34（118.10±33.56)	2587（4541±1298)	49.05（85.23±24.67)
El Salvador	52（89±27)	14.79（25.30±7.83)	158（260±84)	20.14（33.16±10.74)
Equatorial Guinea	1（2±0)	5.53（11.52±2.28)	4（8±1)	8.04（17.33±2.94)
Eritrea	7（14±3)	8.38（16.57±3.84)	18（36±9)	8.16（15.91±3.66)
Estonia	6（10±3)	2.13（3.70±1.15)	18（30±10)	5.04（8.69±2.75)
Eswatini	3（7±2)	12.91（27.07±5.53)	8（14±4)	14.15（26.41±6.63)
Ethiopia	166（301±78)	8.83（16.06±4.15)	296（509±155)	6.85（11.90±3.51)
Fiji	1（1±0)	1.93（4.01±0.83)	2（4±1)	2.68（5.14±1.23)
Finland	52（85±29)	5.51（8.95±3.03)	131（204±77)	7.96（12.51±4.63)
France	910（1504±502)	8.32（13.75±4.61)	1168（1823±668)	6.00（9.50±3.41)
Gabon	6（11±3)	8.99（16.88±4.12)	13（25±6)	12.46（24.33±5.37)
Gambia	2（4±1)	6.13（11.66±2.84)	7（14±3)	7.16（14.41±2.74)
Georgia	70（119±38)	9.01（15.28±4.90)	61（106±32)	7.53（12.99±3.95)
Germany	2008（3223±1133)	11.88（19.05±6.73)	2663（4069±1582)	10.22（15.73±6.08)
Ghana	42（83±19)	7.34（14.68±3.27)	226（424±105)	14.93（27.89±6.85)
Greece	167（266±96)	8.59（13.60±4.93)	196（293±115)	5.55（8.42±3.23)
Greenland	0（0±0)	3.29（6.23±1.61)	0（1±0)	3.17（5.88±1.57)
Grenada	1（2±1)	9.61（16.31±5.27)	2（3±1)	12.61（20.27±7.23)
Guam	1（1±0)	7.92（14.45±4.02)	1（3±1)	5.17（9.22±2.60)
Guatemala	66（111±36)	18.56（31.05±10.15)	320（537±177)	24.69（41.29±13.62)
Guinea	29（57±13)	7.93（15.57±3.52)	45（85±21)	7.75（14.74±3.68)
Guinea-Bissau	4（7±2)	10.00（19.13±4.54)	6（11±3)	9.41（18.31±4.18)
Guyana	9（15±5)	22.24（37.59±11.97)	17（29±9)	22.13（38.12±11.66)
Haiti	60（114±28)	17.90（34.79±7.99)	105（200±47)	14.28（27.52±6.35)
Honduras	34（60±18)	15.49（27.19±8.06)	175（344±66)	24.60（48.36±9.33)
Hungary	148（255±81)	7.51（12.93±4.14)	174（296±96)	6.69（11.49±3.68)
Iceland	1（1±0)	1.51（2.55±0.81)	1（2±1)	1.42（2.36±0.79)
India	1808（3264±933)	3.98（7.22±2.03)	6166（10952±3123)	4.75（8.47±2.37)
Indonesia	845（1685±383)	9.25（18.74±4.09)	2759（4623±1461)	13.13（22.03±6.89)
Iran (Islamic Republic of)	260（456±135)	11.89（21.06±6.12)	929（1488±529)	11.43（18.30±6.49)
Iraq	61（125±27)	6.98（14.23±3.11)	158（293±72)	7.33（13.55±3.37)
Ireland	20（32±11)	3.68（5.90±2.08)	35（54±21)	3.42（5.22±2.02)
Israel	48（76±27)	7.55（12.08±4.27)	91（136±54)	5.41（8.14±3.22)
Italy	2240（3291±1384)	18.77（27.51±11.64)	1757（2456±1101)	7.92（11.18±4.96)
Jamaica	11（19±6)	4.92（8.02±2.77)	19（32±10)	4.94（8.15±2.66)
Japan	1047（1714±593)	5.09（8.34±2.88)	1616（2703±872)	2.64（4.40±1.45)
Jordan	10（19±5)	8.99（17.17±4.26)	47（81±23)	7.41（12.89±3.75)
Kazakhstan	98（170±53)	6.84（11.73±3.72)	444（744±246)	22.97（38.29±12.63)
Kenya	97（200±40)	11.73（24.27±4.87)	381（708±189)	17.98（33.64±8.86)
Kiribati	0（0±0)	5.04（10.66±2.04)	0（1±0)	5.29（10.55±2.38)
Kuwait	3（5±1)	5.48（9.60±2.87)	20（33±11)	8.12（13.58±4.32)
Kyrgyzstan	35（60±18)	10.06（17.42±5.31)	47（82±24)	9.02（15.54±4.71)
Lao People's Democratic Re	9（21±4)	4.60（10.64±1.71)	17（34±8)	3.76（7.67±1.66)
Latvia	11（19±6)	2.38（4.08±1.29)	23（39±12)	4.38（7.45±2.36)
Lebanon	20（37±9)	9.15（17.54±4.16)	70（125±35)	8.41（15.06±4.18)
Lesotho	7（16±2)	7.34（18.53±2.57)	14（26±6)	11.91（23.18±5.51)
Liberia	13（25±7)	11.00（20.52±5.31)	22（42±10)	11.59（22.19±5.39)
Libya	20（55±7)	10.45（28.32±3.42)	72（156±28)	14.75（32.35±5.71)
Lithuania	12（20±6)	2.01（3.48±1.08)	37（64±20)	4.93（8.42±2.67)
Luxembourg	8（12±4)	10.76（17.33±6.13)	10（16±6)	7.22（11.36±4.14)
Madagascar	32（65±14)	6.26（12.71±2.73)	59（121±26)	6.22（12.70±2.72)
Malawi	43（80±21)	11.19（21.22±5.38)	92（169±46)	12.91（23.99±6.21)
Malaysia	25（46±12)	2.51（4.57±1.23)	156（288±74)	5.03（9.31±2.35)
Maldives	0（1±0)	3.18（6.68±1.35)	1（1±0)	2.11（3.94±0.98)
Mali	25（50±12)	6.53（12.92±2.88)	61（120±28)	7.14（14.06±3.25)
Malta	2（4±1)	4.57（7.53±2.49)	5（7±3)	3.47（5.48±1.99)
Marshall Islands	0（0±0)	3.99（8.28±1.64)	0（0±0)	3.93（7.96±1.66)
Mauritania	13（25±6)	12.15（23.91±5.25)	26（51±12)	11.56（22.16±5.36)
Mauritius	3（5±1)	3.43（6.10±1.77)	6（11±3)	2.70（4.75±1.41)
Mexico	1338（2068±779)	29.31（45.16±17.09)	5234（7561±3321)	34.59（49.75±21.97)
Micronesia (Federated State	0（1±0)	4.61（9.40±2.01)	0（1±0)	4.80（9.31±2.11)
Monaco	1（2±0)	9.40（15.93±5.00)	1（2±1)	10.53（16.82±5.88)
Mongolia	19（34±9)	16.95（30.81±8.37)	33（59±17)	16.81（29.77±8.32)
Montenegro	1（2±1)	1.70（3.09±0.84)	3（6±2)	2.51（4.48±1.22)
Morocco	86（174±38)	5.72（11.70±2.48)	326（597±156)	9.10（16.71±4.35)
Mozambique	26（49±12)	4.94（9.64±2.26)	53（103±23)	5.75（11.40±2.34)
Myanmar	60（139±23)	2.25（5.42±0.85)	125（245±55)	2.23（4.39±0.97)
Namibia	4（10±2)	6.74（15.27±2.62)	10（18±5)	7.16（13.74±3.32)
Nauru	0（0±0)	5.35（10.06±2.42)	0（0±0)	5.11（10.47±1.81)
Nepal	33（75±13)	3.57（8.30±1.35)	146（277±68)	5.89（11.19±2.73)
Netherlands	89（144±50)	3.40（5.49±1.90)	181（280±107)	3.73（5.79±2.19)
New Zealand	12（18±7)	2.29（3.47±1.37)	28（39±18)	2.46（3.47±1.55)
Nicaragua	19（32±10)	11.42（19.56±5.98)	91（151±49)	16.24（26.98±8.70)
Niger	21（41±9)	8.11（16.59±3.49)	57（121±25)	7.42（15.70±3.16)
Nigeria	444（826±213)	10.00（18.58±4.78)	767（1324±409)	9.21（15.80±4.94)
Niue	0（0±0)	4.03（7.79±1.88)	0（0±0)	5.15（10.08±2.17)
North Macedonia	5（9±2)	2.11（3.77±1.08)	13（23±6)	3.11（5.58±1.53)
Northern Mariana Islands	0（0±0)	13.13（23.75±6.59)	1（1±0)	12.28（21.30±6.10)
Norway	24（36±14)	2.54（3.94±1.49)	40（58±24)	2.89（4.27±1.77)
Oman	5（10±2)	7.91（16.46±3.38)	18（34±9)	12.35（22.51±5.87)
Pakistan	332（827±112)	5.54（14.04±1.83)	719（1318±326)	6.30（11.62±2.84)
Palau	0（0±0)	4.53（8.67±2.08)	0（0±0)	5.32（10.49±2.29)
Palestine	10（22±4)	10.90（25.08±4.23)	16（29±8)	7.61（13.67±3.73)
Panama	14（23±8)	8.24（13.54±4.47)	51（85±28)	9.23（15.41±5.03)
Papua New Guinea	2（4±1)	1.02（2.17±0.42)	4（8±2)	0.90（1.77±0.41)
Paraguay	7（13±4)	2.82（4.94±1.47)	30（53±15)	4.28（7.59±2.14)
Peru	273（460±150)	20.28（34.13±11.10)	1181（1992±622)	29.09（49.07±15.31)
Philippines	67（139±29)	2.49（5.19±1.05)	236（401±128)	2.77（4.71±1.49)
Poland	232（381±133)	4.22（6.90±2.43)	501（794±298)	5.09（8.12±3.01)
Portugal	244（401±136)	13.47（22.01±7.52)	226（348±132)	6.60（10.27±3.83)
Puerto Rico	111（172±65)	24.57（37.96±14.41)	189（276±118)	18.77（27.67±11.73)
Qatar	2（4±1)	33.17（62.56±15.87)	14（25±6)	30.92（54.97±14.55)
Republic of Korea	132（248±64)	5.00（9.52±2.36)	181（363±82)	1.54（3.11±0.69)
Republic of Moldova	124（213±66)	22.36（38.28±12.04)	178（301±97)	22.13（37.44±12.06)
Romania	260（455±138)	7.33（12.75±3.91)	656（1104±361)	12.94（21.88±7.08)
Russian Federation	784（1302±441)	3.46（5.70±1.97)	2725（4367±1619)	8.28（13.25±4.89)
Rwanda	31（57±15)	11.15（21.02±5.38)	59（121±28)	10.08（20.32±4.62)
Saint Kitts and Nevis	1（2±1)	18.87（30.85±10.72)	1（2±1)	17.96（29.15±10.07)
Saint Lucia	2（3±1)	18.90（31.30±10.70)	5（7±3)	15.65（24.93±8.91)
Saint Vincent and the Grenac	1（1±0)	8.49（14.34±4.61)	2（3±1)	10.57（17.27±5.89)
Samoa	0（1±0)	4.17（8.12±1.87)	1（1±0)	4.06（7.63±1.85)
San Marino	1（1±0)	16.41（27.46±8.63)	1（2±1)	9.75（16.88±4.85)
Sao Tome and Principe	1（2±1)	16.82（32.52±7.88)	2（4±1)	17.68（33.59±7.47)
Saudi Arabia	111（276±40)	21.63（53.98±7.73)	352（619±172)	29.76（51.54±14.64)
Senegal	24（47±12)	7.22（13.97±3.40)	57（113±24)	7.16（14.19±2.99)
Serbia	46（84±24)	3.41（6.32±1.71)	80（143±40)	3.51（6.30±1.75)
Seychelles	0（1±0)	5.86（10.51±2.94)	1（2±0)	7.46（13.45±3.67)
Sierra Leone	16（31±7)	7.34（14.09±3.34)	25（47±12)	6.79（12.71±3.20)
Singapore	6（11±3)	2.57（4.53±1.30)	13（22±7)	1.23（2.11±0.63)
Slovakia	37（65±19)	4.69（8.27±2.45)	67（118±34)	5.23（9.21±2.67)
Slovenia	19（34±10)	6.08（10.60±3.28)	30（51±16)	4.90（8.36±2.63)
Solomon Islands	0（1±0)	3.95（8.41±1.63)	2（3±1)	5.32（10.11±2.48)
Somalia	25（50±11)	12.95（26.12±5.69)	64（128±28)	12.20（24.85±5.31)
South Africa	117（222±57)	5.34（10.12±2.58)	421（677±241)	8.40（13.46±4.78)
South Sudan	27（53±13)	9.67（19.19±4.48)	44（86±21)	12.59（24.82±5.87)
Spain	932（1507±525)	12.87（20.78±7.27)	914（1392±535)	6.63（10.21±3.87)
Sri Lanka	38（75±18)	3.60（7.01±1.66)	74（144±33)	2.32（4.51±1.03)
Sudan	61（154±21)	6.75（17.38±2.24)	147（347±57)	8.10（19.33±3.10)
Suriname	5（8±3)	16.92（28.83±8.80)	12（21±6)	15.97（27.93±7.98)
Sweden	50（82±26)	2.44（4.08±1.28)	87（153±41)	3.01（5.29±1.44)
Switzerland	56（90±31)	4.10（6.70±2.27)	96（150±55)	3.93（6.18±2.26)
Syrian Arab Republic	44（86±20)	8.84（17.23±3.99)	134（250±65)	11.34（21.01±5.48)
Taiwan (Province of China)	70（126±36)	4.36（7.85±2.24)	162（296±80)	2.90（5.29±1.43)
Tajikistan	19（34±10)	6.26（11.23±3.24)	29（57±14)	5.29（10.08±2.49)
Thailand	152（293±71)	4.19（8.09±1.92)	832（1555±399)	6.00（11.21±2.88)
Timor-Leste	1（2±0)	2.76（7.01±0.92)	3（6±1)	2.62（6.00±1.06)
Togo	7（14±3)	6.07（11.80±2.87)	24（48±11)	7.14（14.23±3.20)
Tokelau	0（0±0)	3.72（7.78±1.56)	0（0±0)	3.79（7.10±1.78)
Tonga	1（1±0)	8.55（16.65±3.95)	1（2±0)	9.14（17.71±4.03)
Trinidad and Tobago	12（19±7)	12.43（19.91±7.06)	21（34±12)	8.50（13.57±4.73)
Tunisia	38（77±17)	8.23（16.48±3.55)	158（297±74)	10.77（20.22±5.06)
Turkey	143（283±66)	4.06（8.11±1.86)	503（922±235)	4.75（8.72±2.22)
Turkmenistan	25（44±13)	12.18（21.23±6.54)	95（164±51)	21.43（36.53±11.50)
Tuvalu	0（0±0)	4.04（8.57±1.68)	0（0±0)	4.10（8.00±1.90)
Uganda	44（83±21)	6.93（13.23±3.18)	104（197±46)	7.66（14.68±3.30)
Ukraine	381（640±213)	3.97（6.63±2.24)	549（958±292)	5.16（9.01±2.75)
United Arab Emirates	4（8±2)	13.48（25.23±6.35)	36（65±17)	25.98（46.72±12.57)
United Kingdom	497（729±311)	4.10（6.03±2.56)	1311（1785±866)	7.83（10.71±5.16)
United Republic of Tanzania	90（164±44)	8.07（14.93±3.86)	233（438±108)	9.24（17.45±4.24)
United States of America	1689（2719±970)	3.96（6.40±2.27)	3725（6020±2120)	4.70（7.61±2.67)
United States Virgin Islands	2（3±1)	21.97（35.89±11.94)	4（6±2)	14.31（22.66±8.10)
Uruguay	27（45±15)	5.19（8.70±2.84)	33（54±18)	4.40（7.21±2.41)
Uzbekistan	119（208±64)	8.98（15.58±4.82)	311（546±164)	10.88（18.90±5.75)
Vanuatu	0（1±0)	4.18（10.64±1.37)	1（2±0)	4.34（9.72±1.63)
Venezuela (Bolivarian Repul	133（219±74)	12.31（20.16±6.85)	452（768±238)	12.26（20.80±6.47)
Viet Nam	171（375±69)	3.90（8.61±1.55)	353（679±159)	3.46（6.67±1.54)
Yemen	30（78±10)	7.19（19.44±2.23)	65（135±28)	5.17（10.93±2.13)
Zambia	31（59±15)	11.56（21.64±5.49)	69（133±29)	11.06（21.37±4.56)
Zimbabwe	28（51±14)	6.92（12.80±3.36)	53（106±21)	8.10（16.14±3.11)
