# Supplementary material for: The global impact of non-alcoholic fatty liver disease (including cirrhosis) in the elderly from 1990 to 2021 and future projections of disease burden
Source: PLoS One. 2025 Jun 25;20(6):e0325961. doi: 10.1371/journal.pone.0325961 (PMC12193573; doi:10.1371/journal.pone.0325961)
Supplement: S4 Table — (PDF) [file pone.0325961.s004.pdf]

S4 Table. DALYs of NAFLD among eldly in all countries and regions in 1990 and 2021

	1990		2021	
location	Counts	Age standardized YLDs rate per 100,000 (95%	Counts	Age standardized YLDs rate per 100,000
Afghanistan	1703（3578±663)	216.44（470.00±81.93)	1385（2846±576)	170.98（354.02±70.16)
Albania	162（297±81)	69.27（126.34±34.68)	302（561±143)	51.39（95.22±24.25)
Algeria	1157（2405±479)	94.41（193.53±40.23)	4652（9074±2136)	126.64（247.75±57.16)
American Samoa	3（5±1)	107.69（200.64±52.88)	7（13±4)	127.06（227.05±63.45)
Andorra	13（25±6)	182.72（336.22±88.49)	26（45±13)	135.41（231.98±68.50)
Angola	641（1247±305)	154.54（305.60±71.76)	1720（3312±819)	139.54（269.70±65.84)
Antigua and Barbuda	14（24±8)	200.39（340.62±111.17)	30（48±17)	224.18（361.16±126.98)
Argentina	5510（9457±3016)	132.97（227.92±72.66)	9014（15104±4966)	125.40（210.61±69.02)
Armenia	279（506±145)	85.27（150.87±45.24)	930（1582±508)	160.92（273.27±87.61)
Australia	1695（2753±977)	65.58（106.56±37.78)	4238（6345±2591)	73.00（109.83±44.52)
Austria	3542（5787±1960)	228.70（374.70±126.88)	4024（6231±2379)	177.10（275.85±104.48)
Azerbaijan	1224（2100±638)	225.83（384.90±118.39)	3934（7069±1928)	349.69（625.25±169.26)
Bahamas	58（95±33)	328.20（536.34±188.45)	135（220±77)	274.83（445.14±156.99)
Bahrain	54（99±27)	377.12（687.83±188.72)	246（433±124)	372.46（647.65±187.38)
Bangladesh	5300（9821±2531)	99.68（184.96±47.70)	12611（24139±6096)	78.01（149.25±37.68)
Barbados	80（128±45)	208.56（335.84±119.39)	113（182±62)	162.35（261.37±89.22)
Belarus	710（1235±376)	41.25（71.21±22.03)	2126（3737±1139)	94.53（165.83±50.64)
Belgium	4329（6845±2498)	210.99（333.20±122.17)	5871（8533±3680)	200.63（293.28±125.20)
Belize	28（47±16)	250.29（411.29±138.60)	137（217±79)	396.56（623.50±228.18)
Benin	373（722±171)	160.92（312.80±73.48)	905（1705±443)	165.31（311.53±80.68)
Bermuda	26（40±15)	333.73（514.58±197.99)	32（49±19)	174.82（268.99±103.59)
Bhutan	26（55±11)	99.78（209.12±41.89)	101（197±47)	142.16（275.47±65.67)
Bolivia (Plurinational State of)	2186（4234±849)	588.27（1137.90±229.96)	8491（15597±3883)	771.67（1413.59±353.18)
Bosnia and Herzegovina	433（782±217)	86.90（156.07±43.91)	745（1304±368)	87.72（153.92±43.21)
Botswana	76（162±33)	123.44（264.13±51.69)	202（375±98)	128.70（238.85±62.17)
Brazil	9484（15714±5468)	91.24（150.00±52.68)	38040（58612±23084)	120.04（184.50±72.84)
Brunei Darussalam	5（10±3)	50.66（93.98±23.94)	16（30±8)	42.90（78.92±20.37)
Bulgaria	1666（2952±883)	96.15（169.45±51.14)	2513（4398±1329)	133.73（235.47±70.37)
Burkina Faso	590（1128±284)	121.39（231.28±58.19)	1329（2537±625)	132.52（252.03±62.36)
Burundi	461（880±214)	174.49（334.02±80.25)	661（1237±317)	130.21（243.09±61.61)
Cabo Verde	27（59±11)	92.16（196.96±38.90)	75（143±34)	142.63（273.19±64.15)
Cambodia	962（2222±376)	192.83（457.75±73.68)	2164（4349±980)	154.74（314.40±68.86)
Cameroon	980（1839±466)	200.66（376.68±94.77)	2646（5023±1159)	200.54（379.47±87.21)
Canada	4493（7428±2439)	105.46（174.64±57.19)	14212（22354±8136)	147.77（232.97±84.42)
Central African Republic	175（342±82)	139.39（276.74±64.22)	343（665±159)	151.52（296.04±69.19)
Chad	416（829±181)	126.50（253.18±54.76)	872（1716±411)	141.88（280.62±66.21)
Chile	3873（6368±2167)	311.83（511.92±174.51)	8839（14159±5000)	266.77（427.11±150.99)
China	49935（86874±27031)	52.21（90.68±28.16)	85142（143752±45827)	32.43（55.01±17.38)
Colombia	2115（3525±1154)	104.66（173.92±57.16)	9697（15496±5436)	140.75（224.71±78.94)
Comoros	39（75±19)	178.56（340.47±86.05)	104（197±49)	191.13（364.22±88.47)
Congo	209（394±101)	170.79（324.34±80.99)	508（1003±232)	182.52（357.96±83.10)
Cook Islands	0（0±0)	7.52（14.41±3.39)	0（1±0)	8.73（16.72±3.91)
Costa Rica	437（714±243)	209.70（342.29±116.80)	2395（3724±1387)	343.45（533.33±198.90)
Coted'Ivoire	627（1191±304)	158.22（300.63±76.13)	1686（3210±809)	149.29（283.20±71.20)
Croatia	1075（1883±581)	132.76（231.46±71.41)	1278（2149±696)	107.90（182.02±58.56)
Cuba	2046（3358±1155)	158.73（260.33±89.57)	5305（8240±3097)	220.40（342.89±128.90)
Cyprus	155（276±76)	163.02（294.49±79.57)	225（389±118)	84.83（146.68±44.41)
Czechia	1515（2648±817)	81.42（142.05±44.11)	2874（4921±1591)	106.04（182.51±58.59)
Democratic People's Republic of Korea	796（1621±363)	45.85（94.30±20.48)	1326（2726±578)	33.74（69.38±14.72)
Democratic Republic of the Congo	1863（3572±891)	105.10（203.32±49.64)	3738（7555±1658)	95.46（192.38±41.91)
Denmark	903（1493±503)	88.68（147.44±49.32)	1814（2847±1048)	122.33（192.93±70.59)
Djibouti	17（34±8)	129.79（259.43±60.48)	96（188±44)	153.79（303.37±68.53)
Dominica	22（38±12)	281.55（484.95±148.38)	31（52±16)	287.68（481.96±154.04)
Dominican Republic	1468（2512±790)	355.22（606.04±191.21)	4734（8764±2191)	392.62（726.00±181.87)
Ecuador	3345（5218±1995)	547.91（852.46±326.93)	14344（21381±8749)	712.85（1060.53±435.36)
Egypt	21339（37500±10878)	970.50（1703.13±487.67)	52503（92808±26319)	838.08（1466.23±421.32)
El Salvador	1053（1812±561)	296.62（510.16±157.95)	3065（5061±1634)	402.09（664.64±214.45)
Equatorial Guinea	25（51±11)	113.68（231.85±47.59)	83（176±31)	157.42（335.64±58.80)
Eritrea	164（316±78)	160.43（313.45±74.91)	394（761±185)	148.94（288.33±68.55)
Estonia	127（222±68)	47.04（81.85±25.41)	378（655±206)	114.30（200.12±62.13)
Eswatini	72（143±32)	238.69（484.77±104.64)	168（315±80)	272.61（509.20±128.99)
Ethiopia	3697（6767±1737)	170.42（310.73±80.19)	5903（10112±3136)	126.23（217.17±66.33)
Fiji	16（32±7)	40.31（82.70±17.65)	50（97±23)	53.69（103.27±24.84)
Finland	986（1625±541)	104.89（173.27±57.60)	2707（4265±1583)	175.49（279.17±101.82)
France	17969（30022±9923)	168.84（282.28±93.43)	21341（33955±12186)	120.21（193.92±68.24)
Gabon	119（219±57)	172.26（318.73±81.17)	282（541±124)	238.98（460.57±104.57)
Gambia	44（83±21)	115.88（219.57±54.95)	143（287±58)	134.53（269.56±53.61)
Georgia	1464（2515±794)	180.98（308.03±99.04)	1187（2046±630)	146.80（253.19±77.68)
Germany	37876（61385±21337)	230.38（373.59±130.19)	49515（76606±29350)	205.47（319.67±121.78)
Ghana	871（1684±404)	132.81（260.44±60.68)	4470（8449±2116)	258.81（486.27±120.96)
Greece	2953（4756±1666)	148.32（238.39±83.93)	3174（4831±1849)	101.40（156.10±58.69)
Greenland	3（6±1)	73.76（139.70±36.42)	7（13±4)	71.52（132.32±35.75)
Grenada	18（31±10)	191.17（327.06±104.76)	35（57±20)	250.18（405.31±143.58)
Guam	12（23±6)	146.19（268.07±74.59)	30（54±15)	109.01（194.00±55.04)
Guatemala	1434（2428±782)	361.62（609.04±197.61)	6690（11294±3713)	498.89（840.94±276.67)
Guinea	591（1140±270)	147.88（287.34±66.87)	897（1703±435)	142.85（271.22±68.97)
Guinea-Bissau	86（161±41)	193.23（365.63±89.83)	128（250±59)	175.68（341.40±79.33)
Guyana	186（318±100)	426.34（727.30±228.71)	369（644±194)	457.74（795.44±240.84)
Haiti	1325（2496±615)	352.17（671.71±160.91)	2294（4332±1035)	283.30（539.39±127.18)
Honduras	728（1286±386)	307.92（543.16±162.80)	3667（7223±1411)	485.07（954.32±186.42)
Hungary	3316（5769±1817)	165.69（288.20±90.99)	3798（6530±2107)	150.69（261.11±83.07)
Iceland	11（19±6)	31.27（52.87±16.80)	21（36±12)	28.34（47.13±15.65)
India	39691（71573±20614)	77.29（139.56±39.86)	123803（218904±63797)	87.55（155.16±44.65)
Indonesia	17379（34268±8008)	168.21（335.36±76.28)	54059（91203±28691)	219.31（368.48±115.82)
Iran (Islamic Republic of)	5215（9095±2716)	197.95（348.28±102.44)	16206（26117±9218)	188.04（301.89±106.82)
Iraq	1155（2323±525)	128.98（259.78±58.55)	3055（5699±1371)	125.89（233.66±57.11)
Ireland	381（617±214)	70.39（114.24±39.59)	711（1088±420)	70.69（108.45±41.69)
Israel	898（1444±503)	139.81（224.89±78.68)	1559（2361±929)	96.01（146.38±56.93)
Italy	43029（63942±26641)	360.72（535.74±223.95)	27158（38391±17055)	136.28（194.57±85.40)
Jamaica	224（365±126)	97.49（159.37±54.93)	374（625±201)	96.54（161.06±51.84)
Japan	19608（32431±11110)	91.49（151.06±51.97)	23177（38567±12793)	44.98（75.19±25.12)
Jordan	195（369±94)	153.63（291.28±73.57)	873（1533±438)	121.43（212.14±61.31)
Kazakhstan	2005（3531±1079)	132.56（229.76±72.18)	9050（15350±5010)	422.94（712.22±233.64)
Kenya	1940（3999±811)	214.51（442.47±89.57)	7794（14410±3886)	321.63（597.20±159.57)
Kiribati	4（9±2)	104.31（216.99±43.81)	9（17±4)	106.40（210.09±49.01)
Kuwait	52（92±27)	95.84（168.66±50.54)	335（570±178)	130.77（221.00±69.67)
Kyrgyzstan	705（1231±369)	192.84（334.68±101.94)	1034（1813±534)	180.83（313.77±93.99)
Lao People's Democratic Republic	206（442±84)	87.44（194.20±34.39)	345（695±156)	68.90（139.02±30.92)
Latvia	247（425±133)	52.42（89.85±28.38)	486（831±261)	97.55（167.53±52.35)
Lebanon	352（661±163)	148.95（281.85±68.54)	1028（1842±509)	129.59（232.56±64.07)
Lesotho	132（319±50)	134.73（329.94±49.61)	292（566±141)	230.03（446.53±108.85)
Liberia	275（509±136)	202.67（376.69±99.04)	444（854±207)	213.19（409.45±99.45)
Libya	361（946±124)	175.78（464.30±59.91)	1284（2727±525)	247.71（529.81±100.36)
Lithuania	261（455±140)	44.09（76.78±23.80)	791（1362±428)	109.36（188.74±59.38)
Luxembourg	150（244±85)	211.76（344.55±120.01)	186（295±107)	139.67（222.35±79.88)
Madagascar	682（1351±306)	118.92（237.84±52.73)	1299（2631±572)	116.06（235.76±51.01)
Malawi	917（1721±457)	211.85（399.54±103.83)	1909（3503±972)	240.08（442.57±119.49)
Malaysia	519（938±258)	49.69（89.61±24.75)	3062（5671±1466)	90.53（167.46±43.02)
Maldives	6（11±2)	60.35（124.09±25.98)	12（22±6)	35.44（65.99±16.55)
Mali	549（1080±253)	123.28（243.14±55.91)	1295（2533±603)	134.28（263.03±62.01)
Malta	50（83±27)	90.61（150.05±49.46)	90（143±52)	68.91（110.21±39.58)
Marshall Islands	2（3±1)	82.98（169.81±34.70)	3（6±1)	79.77（161.75±34.45)
Mauritania	254（488±115)	223.31（433.80±99.68)	520（1008±245)	210.74（406.27±98.89)
Mauritius	57（102±30)	65.05（116.24±33.74)	124（221±65)	51.27（91.06±26.80)
Mexico	27319（42816±15774)	560.66（874.86±324.65)	108550（159134±68786)	697.00（1017.62±442.01)
Micronesia (Federated States of)	6（12±3)	95.42（192.90±42.15)	9（17±4)	98.11（188.92±43.68)
Monaco	17（29±9)	181.45（308.98±97.42)	26（41±14)	200.56（320.80±112.34)
Mongolia	376（681±189)	315.17（571.72±157.58)	633（1144±320)	284.75（509.54±143.05)
Montenegro	30（55±15)	37.56（68.68±18.76)	71（127±35)	52.44（93.85±25.41)
Morocco	1570（3145±706)	97.24（195.84±43.32)	5942（10926±2828)	152.02（278.93±72.49)
Mozambique	511（977±240)	84.58（163.03±39.31)	1059（2038±470)	98.16（191.13±42.03)
Myanmar	1384（3179±545)	47.90（112.04±18.63)	2767（5396±1239)	45.79（89.51±20.36)
Namibia	94（204±39)	125.41（276.95±50.22)	199（379±94)	132.22（252.47±62.15)
Nauru	1（1±0)	110.18（207.10±50.21)	1（1±0)	104.21（213.35±37.30)
Nepal	717（1595±280)	68.87（155.96±26.56)	2898（5487±1368)	105.97（200.85±49.74)
Netherlands	1636（2667±917)	62.96（102.96±35.29)	3220（5042±1896)	68.86（108.39±40.47)
New Zealand	243（372±145)	46.94（72.20±28.03)	536（759±339)	48.41（68.65±30.52)
Nicaragua	382（662±203)	220.05（379.64±116.53)	1905（3171±1025)	328.66（546.46±176.95)
Niger	444（879±197)	150.27（301.87±65.99)	1209（2557±521)	136.19（287.53±58.63)
Nigeria	8721（16311±4189)	177.40（330.99±85.15)	15030（26224±7991)	161.79（279.68±86.58)
Niue	0（0±0)	81.39（156.21±38.57)	0（1±0)	101.51（198.60±43.27)
North Macedonia	107（190±55)	45.02（80.34±23.22)	279（506±139)	62.40（112.80±30.95)
Northern Mariana Islands	4（7±2)	251.96（454.70±128.34)	13（23±7)	230.88（397.99±115.98)
Norway	457（711±269)	51.46（80.56±30.16)	724（1076±442)	55.84（83.56±34.02)
Oman	96（199±42)	142.30（294.43±61.46)	362（673±174)	209.58（384.67±101.00)
Pakistan	6593（16105±2277)	102.18（252.09±34.78)	14389（26406±6574)	112.92（207.25±51.36)
Palau	1（2±1)	91.50（173.93±42.33)	3（6±1)	104.04（205.14±45.70)
Palestine	172（389±69)	179.07（407.33±70.78)	300（538±147)	122.59（219.82±60.18)
Panama	263（435±143)	149.70（247.45±81.34)	990（1661±537)	181.06（303.75±98.23)
Papua New Guinea	42（88±18)	20.64（43.47±8.52)	96（188±44)	18.01（35.41±8.27)
Paraguay	143（251±75)	54.31（95.38±28.47)	604（1084±302)	84.76（151.62±42.37)
Peru	5463（9229±3006)	394.10（664.69±216.76)	22458（38291±11816)	553.68（943.88±291.33)
Philippines	1335（2744±584)	43.47（89.86±18.79)	4789（8173±2601)	51.28（87.31±27.79)
Poland	4570（7565±2621)	80.27（132.34±46.19)	11020（17689±6513)	113.03（182.35±66.37)
Portugal	4954（8201±2760)	265.01（438.06±147.94)	4016（6276±2325)	128.09（202.17±73.83)
Puerto Rico	2149（3372±1252)	466.07（732.10±271.80)	3486（5176±2174)	372.18（557.70±231.21)
Qatar	37（69±18)	537.32（1007.61±258.04)	273（505±131)	496.39（891.32±236.68)
Republic of Korea	2601（4891±1267)	85.22（161.22±40.95)	2915（5801±1348)	24.02（47.93±11.07)
Republic of Moldova	2830（4892±1522)	481.78（829.04±259.77)	3920（6691±2130)	478.93（816.47±260.11)
Romania	5679（9957±3012)	153.29（267.46±81.94)	13432（22734±7421)	272.00（462.79±149.24)
Russian Federation	16779（28081±9456)	70.44（116.78±40.11)	60135（97588±35622)	178.60（289.47±105.32)
Rwanda	667（1245±327)	210.78（395.03±102.63)	1227（2519±580)	180.88（368.04±84.44)
Saint Kitts and Nevis	19（32±11)	361.11（597.82±205.32)	31（52±17)	353.97（583.36±197.00)
Saint Lucia	37（62±21)	337.71（567.92±190.67)	86（138±48)	286.38（461.99±161.36)
Saint Vincent and the Grenadines	15（25±8)	161.90（275.47±88.16)	38（63±21)	205.17（338.30±114.17)
Samoa	9（17±4)	84.34（162.61±38.45)	14（26±6)	80.86（150.81±37.27)
San Marino	13（22±7)	277.52（462.09±146.46)	17（29±9)	170.47（298.81±85.91)
Sao Tome and Principe	23（44±11)	295.39（564.77±140.41)	37（70±16)	313.47（594.80±134.82)
Saudi Arabia	2029（4977±729)	357.68（882.15±128.70)	7038（12535±3411)	482.20（844.16±235.73)
Senegal	506（971±242)	136.66（262.90±65.02)	1151（2277±505)	132.31（261.45±57.15)
Serbia	1026（1894±534)	68.97（127.55±35.27)	1646（2977±828)	73.11（132.60±36.64)
Seychelles	8（14±4)	107.91（192.56±54.63)	17（32±9)	128.07（233.55±63.64)
Sierra Leone	321（612±150)	134.78（257.93±62.45)	508（947±244)	124.94（233.22±59.80)
Singapore	131（232±67)	51.14（90.29±25.94)	233（401±122)	21.50（37.02±11.20)
Slovakia	813（1433±425)	101.14（178.35±52.94)	1457（2565±750)	113.00（199.38±57.78)
Slovenia	425（749±228)	133.61（234.18±72.00)	556（949±300)	95.44（163.74±51.41)
Solomon Islands	11（24±5)	76.24（161.58±31.66)	36（68±17)	101.01（191.34±47.52)
Somalia	544（1088±245)	248.87（498.48±111.39)	1436（2868±640)	231.95（467.89±102.23)
South Africa	2253（4258±1107)	95.45（180.52±46.69)	8601（13960±4964)	156.96（253.65±90.15)
South Sudan	556（1098±266)	183.64（363.36±86.66)	927（1814±441)	239.46（469.15±113.09)
Spain	17878（29170±10051)	244.35（398.62±137.49)	15499（23913±9050)	124.16（193.36±72.25)
Sri Lanka	763（1494±347)	64.30（125.58±29.40)	1405（2745±624)	40.89（79.84±18.19)
Sudan	1166（2863±423)	115.16（287.11±40.29)	2711（6296±1066)	137.25（321.16±53.63)
Suriname	100（171±53)	332.17（567.33±175.15)	246（428±123)	314.60（548.34±158.01)
Sweden	951（1588±503)	49.34（83.22±26.10)	1622（2844±779)	60.65（107.05±29.56)
Switzerland	1105（1813±611)	85.19（140.10±47.17)	1753（2762±1004)	77.56（122.54±44.48)
Syrian Arab Republic	784（1506±359)	143.91（276.96±65.75)	2478（4654±1188)	180.45（336.75±86.89)
Taiwan (Province of China)	1417（2538±742)	76.71（137.45±39.89)	2761（5025±1375)	49.20（89.63±24.44)
Tajikistan	379（685±193)	118.11（212.41±60.76)	635（1237±307)	100.17（191.59±48.14)
Thailand	3177（6055±1493)	80.12（153.07±37.48)	15654（29236±7578)	111.01（207.22±53.76)
Timor-Leste	14（34±5)	52.84（131.17±18.22)	53（119±22)	49.46（112.51±20.44)
Togo	149（285±72)	114.52（219.73±54.82)	520（1033±238)	132.58（263.21±60.34)
Tokelau	0（0±0)	75.55（155.17±32.06)	0（0±0)	74.90（139.58±35.57)
Tonga	11（22±5)	168.15（325.57±79.02)	17（32±8)	174.81（336.72±78.25)
Trinidad and Tobago	238（388±135)	232.58（377.94±132.12)	457（734±252)	174.94（280.80±96.59)
Tunisia	717（1424±318)	133.01（264.77±58.36)	2711（5124±1267)	172.63（325.51±80.84)
Turkey	2744（5317±1268)	71.88（140.72±33.12)	8647（15799±4065)	77.23（141.35±36.25)
Turkmenistan	524（931±279)	235.61（413.90±126.54)	2101（3647±1113)	432.78（743.83±231.27)
Tuvalu	1（1±0)	83.52（173.33±35.87)	1（2±1)	82.60（160.61±39.07)
Uganda	906（1703±426)	127.44（241.12±59.34)	2071（3913±932)	137.42（260.67±61.08)
Ukraine	8523（14466±4763)	86.31（145.38±48.68)	12786（22375±6787)	119.55（209.31±63.45)
United Arab Emirates	83（158±39)	251.58（472.16±119.32)	794（1425±380)	433.95（779.03±209.26)
United Kingdom	10206（15146±6340)	86.53（129.14±53.62)	26469（36263±17399)	167.20（229.95±109.52)
United Republic of Tanzania	1919（3483±955)	153.92（281.63±75.43)	4684（8813±2217)	171.06（321.89±80.29)
United States of America	34152（55341±19612)	81.61（132.81±46.77)	75734（123335±43264)	96.77（157.98±55.18)
United States Virgin Islands	39（65±21)	409.18（672.65±223.43)	69（110±39)	275.60（439.91±155.31)
Uruguay	516（866±282)	99.47（166.96±54.51)	618（1016±339)	87.27（143.65±47.78)
Uzbekistan	2511（4424±1337)	183.47（320.05±98.66)	7115（12604±3766)	226.87（396.66±120.43)
Vanuatu	6（15±2)	85.60（214.67±28.92)	18（39±7)	88.88（196.96±33.62)
Venezuela (Bolivarian Republic of)	2766（4572±1538)	245.58（404.29±136.86)	9543（16275±4990)	248.80（424.02±130.40)
Viet Nam	3107（6653±1280)	65.84（142.18±26.94)	6340（12090±2888)	57.17（109.22±25.89)
Yemen	597（1495±212)	121.45（315.79±40.59)	1232（2527±542)	86.78（180.20±37.23)
Zambia	662（1243±318)	218.85（409.84±104.80)	1434（2748±625)	206.46（396.19±88.37)
Zimbabwe	566（1050±281)	125.57（232.72±61.58)	1131（2259±443)	151.92（302.96±59.01)
